# Plant Secondary Metabolites as Modulators of Mitochondrial Health: An Overview of Their Anti-Oxidant, Anti-Apoptotic, and Mitophagic Mechanisms

**DOI:** 10.3390/ijms26010380

**Published:** 2025-01-04

**Authors:** Julia Anchimowicz, Piotr Zielonka, Slawomir Jakiela

**Affiliations:** Department of Physics and Biophysics, Institute of Biology, Warsaw University of Life Sciences, 02-787 Warsaw, Poland; julia_anchimowicz@sggw.edu.pl (J.A.); piotr_zielonka@sggw.edu.pl (P.Z.)

**Keywords:** plant secondary metabolites, alkaloids, terpenoids, polyphenols, saponins, mitochondrial dysfunction, cardiovascular diseases, neurodegenerative diseases, metabolic diseases, side effects

## Abstract

Plant secondary metabolites (PSMs) are a diverse group of bioactive compounds, including flavonoids, polyphenols, saponins, and terpenoids, which have been recognised for their critical role in modulating cellular functions. This review provides a comprehensive analysis of the effects of PSMs on mitochondrial health, with particular emphasis on their therapeutic potential. Emerging evidence shows that these metabolites improve mitochondrial function by reducing oxidative stress, promoting mitochondrial biogenesis, and regulating key processes such as apoptosis and mitophagy. Mitochondrial dysfunction, a hallmark of many pathologies, including neurodegenerative disorders, cardiovascular diseases, and metabolic syndrome, has been shown to benefit from the protective effects of PSMs. Recent studies show that PSMs can improve mitochondrial dynamics, stabilise mitochondrial membranes, and enhance bioenergetics, offering significant promise for the prevention and treatment of mitochondrial-related diseases. The molecular mechanisms underlying these effects, including modulation of key signalling pathways and direct interactions with mitochondrial proteins, are discussed. The integration of PSMs into therapeutic strategies is highlighted as a promising avenue for improving treatment efficacy while minimising the side effects commonly associated with synthetic drugs. This review also highlights the need for future research to elucidate the specific roles of individual PSMs and their synergistic interactions within complex plant matrices, which may further optimise their therapeutic utility. Overall, this work provides valuable insights into the complex role of PSMs in mitochondrial health and their potential as natural therapeutic agents targeting mitochondrial dysfunction.

## 1. Introduction

Mitochondria are vital cellular organelles responsible for energy production, regulation of redox status, calcium homeostasis, and initiation of apoptotic pathways [1]. As primary sites of reactive oxygen species (ROS) generation, mitochondria are particularly susceptible to oxidative damage, which is closely linked to cellular ageing and various degenerative diseases, such as Alzheimer’s, Parkinson’s, diabetes, and obesity [2,3,4]. Therefore, the modulation of mitochondrial health represents a promising therapeutic intervention strategy, particularly in these age-related and chronic conditions [2,5,6].

Recent research has identified plant secondary metabolites (PSMs)—bioactive compounds produced by plants that are not involved in their growth, development, or reproduction—as significant modulators of mitochondrial activity [7,8]. These metabolites, which include polyphenols, terpenoids, alkaloids, and glucosinolates, exhibit diverse biological activities, such as anti-oxidant, anti-apoptotic, and anti-inflammatory effects, which can help to preserve mitochondrial integrity and function. This review aims to provide a comprehensive overview of the multiple roles that PSMs play in improving mitochondrial function. Unlike conventional pharmaceuticals, PSMs offer unique advantages due to their evolutionary compatibility with human physiology and their ability to target multiple pathways simultaneously.

**Figure 1:**
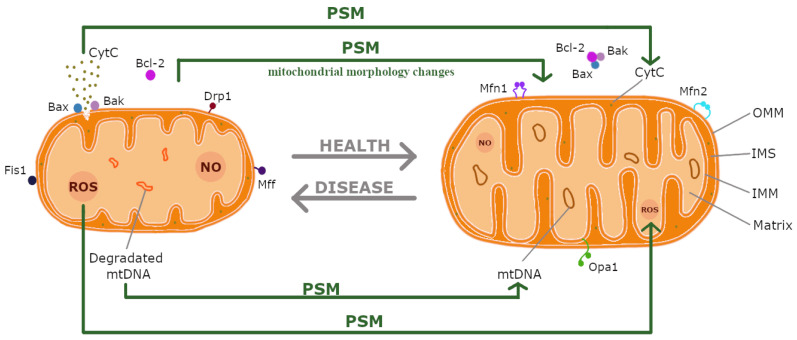
Effects of plant secondary metabolites (PSMs) on disease mechanisms. These compounds: (i) enhance mitochondrial biogenesis, (ii) reduce oxidative stress, (iii) regulate apoptosis and mitophagy, and (iv) alter mitochondrial morphology. Abbreviations: CytC, cytochrome c; Drp1, dynamin-related protein 1; Fis1, mitochondrial fission 1 protein; IMM, inner mitochondrial membrane; IMS, intermembrane space; Mff, mitochondrial fission factor; Mfn1/2, mitofusins 1/2; mtDNA, mitochondrial DNA; NO, nitric oxide; OMM, outer mitochondrial membrane; Opa1, optic atrophy 1 protein; ROS, reactive oxygen species.

The objectives of this review are threefold: first, to elucidate the mechanisms by which PSMs exert their beneficial effects on mitochondrial dynamics, including their anti-oxidant properties and influence on oxidative stress, apoptosis, biogenesis, and mitophagy (Figure 1); second, to assess the therapeutic potential of these compounds in the context of mitochondrial dysfunction; and third, to highlight the need for further research to explore the safety profiles and clinical applications of PSMs in modern medicine.

By integrating traditional phytochemical knowledge with contemporary pharmacological research, this review aims to pave the way for innovative therapeutic approaches that utilise the strengths of natural products in promoting mitochondrial health and treating related pathologies. Furthermore, practical insights into commonly employed concentrations in experimental settings and suitable solvents for metabolite extraction are provided to support ongoing research and application in pharmacology (refer to Supplementary Information, Table S1).

## 2. Mitochondria

### 2.1. Mitochondria: Functions and Structure

Mitochondria are organelles recognised as the powerhouses of eukaryotic cells. Their primary function is to facilitate oxidative phosphorylation (OXPHOS), a process that generates adenosine triphosphate (ATP), the cellular energy currency. ATP is analogous to fuel for a vehicle, driving the metabolic processes by supporting key biochemical reactions [9]. Additionally, mitochondria play crucial roles in the β-oxidation of fatty acids, regulation of intracellular calcium levels (Ca^2^^+^), participation in the tricarboxylic acid (TCA) cycle, and regulation of apoptosis [10]. The mitochondrial apoptosis pathway involves Bcl-2 family proteins and cytochrome C (CytC). Pro-apoptotic proteins, such as Bax and Bak, translocate to the mitochondria upon activation, ultimately leading to programmed cell death. Conversely, Bcl-2 acts as an anti-apoptotic factor by binding to Bax and Bak, thereby inhibiting apoptosis [11]. Following the activation of the apoptotic machinery, CytC is released from the mitochondria into the cytosol, which triggers the activation of the caspase cascade in the intrinsic apoptotic pathway [12].

Mitochondria are thought to have originated from an endosymbiotic event in which an ancestral α-proteobacterium was incorporated into a host cell by endocytosis. This event led to the formation of a double-membraned organelle that contained its own genetic material—mitochondrial DNA (mtDNA) [13]. Over evolutionary time, a significant proportion of the α-proteobacterial genome has been transferred to the nuclear genome of the host cell. Currently, mtDNA, which exists in a plasmid-like structure, encodes only 13 proteins essential for OXPHOS [14].

The functional capabilities of mitochondria are closely linked to their structural organisation. Mitochondria are surrounded by the outer mitochondrial membrane (OMM) and the inner mitochondrial membrane (IMM). The OMM allows molecules up to 10 kDa to pass through. The intermembrane space (IMS), located between the OMM and IMM, is the site of many vital metabolic processes. The IMM is invaginated into structures known as cristae, which define the mitochondrial matrix—a compartment containing the mitochondrial genetic system and the site of bio-synthetic reactions. The IMM is the functional core of mitochondria, housing the electron transport chain (ETC), respiratory enzyme complexes I-IV (in humans), and ATP synthase [14].

### 2.2. Mitochondrial Dynamics

**Figure 2:**
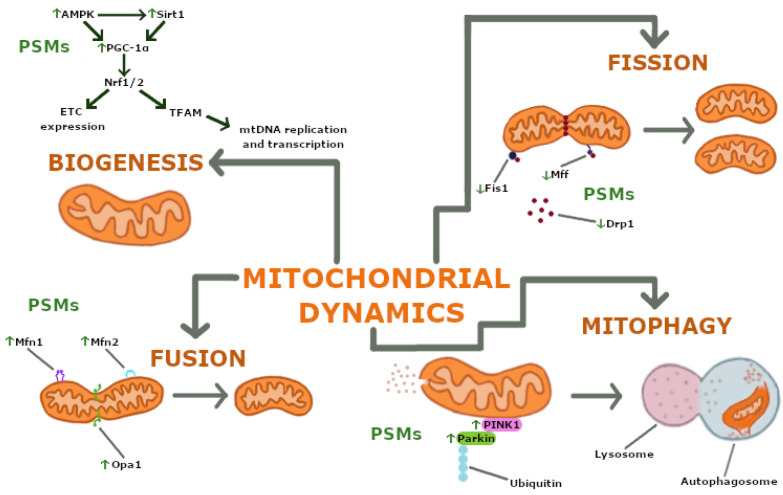
Key processes and associated proteins involved in mitochondrial dynamics: (i) biogenesis, mediated by PGC-1α (peroxisome proliferator-activated receptor gamma coactivator 1α), Nrf2 (nuclear factor erythroid 2-related factor 2), TFAM (mitochondrial transcription factor A), AMPK (AMP-activated protein kinase), and Sirt1 (sirtuin 1); (ii) fusion, regulated by Mfn1 and Mfn2 (mitofusins 1 and 2) and Opa1 (optic atrophy protein 1); (iii) fission, controlled by Drp1 (dynamin-related protein 1), Fis1 (mitochondrial fission protein 1), and Mff (mitochondrial fission factor); and (iv) mitophagy, involving PINK1 (PTEN-induced putative kinase 1) and Parkin. Green arrows indicate increased/decreased expression of key proteins caused by PSMs.

Mitochondria are highly dynamic organelles that can change their structure and function in response to external stimuli. The term “mitochondrial dynamics” encompasses the ongoing processes of mitochondrial biogenesis, fusion, fission, and mitophagy [10,15,16] (Figure 2).

Mitochondrial proliferation occurs exclusively through the growth and division of existing organelles, as de novo synthesis is not possible. Mitochondrial biogenesis encompasses three coordinated processes: mtDNA replication, synthesis of mitochondrial proteins, and generation of the inner and outer mitochondrial membranes (IMM and OMM). These processes are regulated by transcriptional regulators, such as peroxisome proliferator-activated receptor gamma coactivator 1α (PGC-1α) and nuclear factor erythroid 2-related factor 2 (Nrf2), and upstream regulatory molecules. PGC-1α serves as a key regulator of mitochondrial biogenesis, affecting almost all processes associated with this phenomenon. The activation of PGC-1α—stimulated by factors such as exercise or fasting—facilitates its translocation to the nucleus, where it co-activates Nrf2. This translocation initiates the upregulation of ETC protein expression and stimulates mitochondrial transcription factor A (TFAM), which is responsible for mtDNA replication and transcription. Upstream regulatory molecules, including AMP-activated protein kinase (AMPK) and Sirtuin 1 (Sirt1), also contribute to this regulatory network. The activation of AMPK in response to low energy levels stimulates PGC-1α, which subsequently activates the Nrf2/TFAM signalling pathway, leading to increased mitochondrial biogenesis. In addition, Sirt1 promotes the deacetylation of PGC-1α, thereby increasing its activity and improving mitochondrial quality and biogenesis [17].

Mitochondrial fusion occurs when two organelles fuse to form a single mitochondrion. Key proteins involved in this process include mitofusins 1 and 2 (Mfn1/2), located on the OMM, and optic atrophy protein 1 (Opa1), located on the IMM. The activation of these proteins leads to the fusion of the OMM, followed by the fusion of the IMM, ultimately resulting in the fusion of the matrix components [15,16]. In contrast, mitochondrial fission refers to the division of a single mitochondrion into two separate entities. This process is mediated by dynamin-related protein 1 (Drp1), which is found abundantly in the cytosol, and mitochondrial fission protein 1 (Fis1) and mitochondrial fission factor (Mff), both of which are localised to the OMM. The process is initiated by the recruitment of Drp1 from the cytosol to the OMM [16,17]. Mitophagy is an evolutionarily conserved form of autophagy that selectively removes dysfunctional or excess mitochondria. This process can be regulated by different metabolic pathways [15]. Mitophagy involves four sequential steps: depolarisation of the mitochondrial membrane potential (∆Ψm), formation of mitochondrial autophagosomes, fusion of these autophagosomes with lysosomes, and degradation of mitochondrial contents [18]. Numerous mitophagy pathways have been identified, including the PINK1/Parkin-mediated pathway. The serine/threonine PTEN-induced putative kinase 1 (PINK1), encoded by the *PINK1* gene, and the E3 ubiquitin ligase Parkin, encoded by the *PARK2* gene, are critical components of this pathway. PINK1 acts as a mitochondrial damage sensor, Parkin as a signal amplifier, and ubiquitin chains as signal effectors assembled on mitochondria [19]. The accumulation of PINK1 on the OMM of damaged mitochondria facilitates the recruitment of Parkin from the cytosol, enhancing the ubiquitination of mitochondrial proteins and ultimately promoting mitochondrial degradation [20].

These tightly regulated processes are fundamental for cellular function. Their dysregulation contributes to various diseases, making them important therapeutic targets.

### 2.3. Mitochondrial Dysfunctions and Associated Diseases

Mitochondrial dysfunction manifests in multiple diseases, particularly affecting tissues with high energy demands. Cardiovascular, hepatic, neurological, metabolic, and neoplastic disorders each display characteristic patterns of mitochondrial impairment. This section examines these disease-specific mitochondrial alterations.

Cardiovascular disease includes a number of conditions associated with mitochondrial dysfunction. The heart, which is characterised by high energy requirements, is predominantly composed of mitochondria-rich cardiomyocytes. Proper mitophagy in these cells is crucial for maintaining cardiac homeostasis by removing dysfunctional mitochondria, thereby preventing inflammation and other metabolic disorders [21]. Diseases such as cardiomyopathy, heart failure, and ischaemia/reperfusion (I/R) injury are associated with mitochondrial dynamics, with altered expression levels of key proteins involved in fusion and fission processes. These conditions often involve increased ROS, impaired calcium homeostasis, and reduced energy output. I/R injury, characterised by sudden occlusion of blood flow followed by reperfusion, can lead to irreversible tissue damage, necrosis, and mitochondrial dysfunction, which further exacerbates ROS generation [10]. Interestingly, Drp1 inhibition has been shown to attenuate ischaemic brain injury, suggesting that the regulation of mitochondrial dynamics may confer protective effects in this context [17].

Like cardiac tissue, the liver contains cells rich in mitochondria, reflecting its high metabolic demands. In non-alcoholic fatty liver disease (NAFLD), abnormal lipid accumulation leads to impaired mitophagy and altered levels of Drp1 and Fis1 proteins [17,19]. In addition, NAFLD is associated with a significant downregulation of PGC-1α, which contributes to reduced mitochondrial biogenesis [17].

Neuronal tissues have high mitochondrial density, and neurodegenerative diseases (NDs) are characterised by mitochondrial dysfunction and age-related ATP depletion, ultimately resulting in cell death. Impaired mitophagy plays a critical role in these processes. Parkinson’s disease (PD), characterised by the degeneration of dopaminergic neurons in the substantia nigra, is an example of an ND in which the downregulation of PINK1 and Parkin proteins leads to impaired mitophagy and increased oxidative stress [18]. PD is characterised by the aggregation of Lewy bodies, mainly composed of α-synuclein protein [22]. Alzheimer’s disease (AD) is characterised by the accumulation of β-amyloid plaques and tau protein, leading to dementia and cognitive impairment. The downregulation of PGC-1α, defective calcium handling, and reduced levels of Parkin have been observed in AD [17,18,23]. In Huntington’s disease (HD), an autosomal dominant disorder characterised by chorea and dystonia, mutant huntingtin protein (mHtt) interacts with Drp1, enhancing its activity and contributing to changes in mitochondrial structure and mtDNA damage [18].

Mitochondrial dysfunction is implicated in diabetes through complex but incompletely understood mechanisms. As central regulators of energy metabolism, impaired mitochondrial content, biogenesis, and function lead to increased ROS production, disrupting both energy homeostasis and insulin sensitivity [24]. Diabetic retinopathy is characterised by the downregulation of Mfn2, alterations in mitochondrial dynamics, morphology, and ∆Ψm, and cytosolic leakage of CytC [25]. In diabetic nephropathy, alterations in the ETC increase ROS levels and promote mitochondrial fission, as well as reduce PGC-1α levels and ATP production [26].

Cancer progression is characterised by mitochondrial dysfunction, with particularly significant alterations in mitophagy. Carcinogenesis is known to be associated with the accumulation of defective mitochondria [18]. This mitochondrial accumulation in the early stages of tumourigenesis results from impaired mitophagy and promotes tumour growth. Conversely, in advanced cancer, proper mitophagy becomes critical for cancer cell survival [21].

## 3. Plant Secondary Metabolites

**Figure 3:**
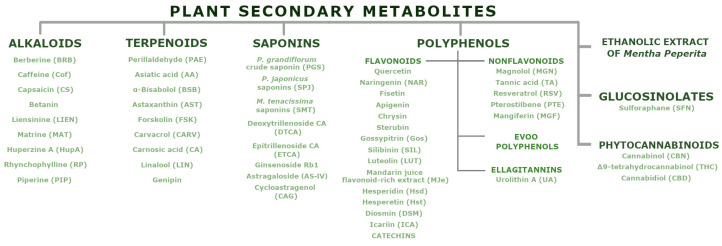
Overview of natural compounds. The primary groups of PSMs discussed in this review include alkaloids (heterocyclic alkaloids), terpenoids, saponins, polyphenols (flavonoids, non-flavonoids, extra virgin olive oil (EVOO) polyphenols, ellagitannins, catechins), glucosinolates, and phytocannabinoids.

PSMs are low-molecular-weight organic compounds that mediate plant–environment interactions but are not involved in primary growth and development. These compounds, categorised by their structural characteristics and properties [27], demonstrate effects on biological processes, including mitochondrial function. Figure 3 presents the natural compounds discussed in this review.

### 3.1. Alkaloids

**Figure 4:**
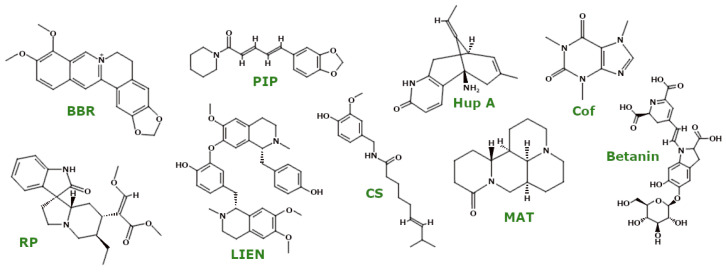
Chemical structures of berberine (BRB), caffeine (Cof), capsaicin (CS), betanin, liensinine (LIEN), matrine (MAT), huperzine A (Hup A), rhynchophylline (RP), and piperine (PIP).

Alkaloids (Figure 4) are a diverse group of phytochemicals consisting primarily of carbon, hydrogen, nitrogen, and usually oxygen. These compounds are predominantly found in flowering plants [28]. Among them is berberine (BBR), known for its distinctive yellow colour and bitter taste. Um et al. investigated the effects of BBR on BEAS-2B cells (human non-tumourigenic lung epithelial cell line), HeLa Parkin cells, and A549 cells (human lung adenocarcinoma cell line). Their results showed that BBR specifically induced mitophagy without affecting mitochondrial function. At the same time, BBR enhanced mitochondrial biogenesis by increasing the expression levels of PGC1α and Nrf1, which are key regulators of this process. Furthermore, in PINK1 knockout mouse embryonic fibroblasts (PINK1 KO MEFs), BBR treatment ameliorated mitochondrial dysfunction by reversing the decrease in mitochondrial respiration and ΔΨm while reducing elevated levels of mitochondrial ROS [29].

Berberine’s protective effects against D-ribose-induced ageing were investigated by Wang et al. in both Neuro2a cells (murine neuroblastoma cell line) and APP/PS1 mice, a transgenic model of AD. In their study, D-ribose acted as an ageing inducer, causing a decrease in ΔΨm, increased ROS, CytC release, and disruptions in the PINK1/Parkin mitophagy pathway. Notably, BBR treatment alleviated these impairments by increasing ΔΨm and reducing ROS and CytC release. In addition, BBR promoted mitophagy by inhibiting the methylation of the PINK1 promoter, thereby inducing its expression [30]. Researchers have also found that BBRP, a fluorescently labelled derivative of BBR, can downregulate the accumulation of PINK1 in PC12 cells (rat adrenal medullary pheochromocytoma cell line), thereby regulating the mitophagy process [31].

Berberine regulates mitochondrial complex activity, as demonstrated across several studies. Specifically, Phogat et al. found that berberine pretreatment prevented acetamiprid (ACMP)-induced loss of mitochondrial complex activity in rats, where ACMP is known to trigger oxidative stress and mitochondrial dysfunction. BBR alleviated these symptoms by increasing the activity of mitochondrial complexes [32]. In a study by Yadawa et al., rats treated with D-galactose to induce ageing showed increased levels of malondialdehyde (MDA), a pro-oxidant, and decreased levels of reduced glutathione (GSH), an anti-oxidant. D-galactose also impaired the activity of mitochondrial complexes I, II, III, and IV. BBR treatment counteracted these changes by restoring redox balance and increasing mitochondrial complex activity [33].

Berberine pretreatment showed positive effects in a rotenone (RTN)-induced model of PD, as reported by Tseng et al. Administration of BBR prior to RTN treatment resulted in reduced levels of nitric oxide (NO) and lipid peroxidation (LPO), as well as increased activity of mitochondrial enzymes, including succinate dehydrogenase (SDH), ATPase, and proteins involved in the ETC. Taken together, these findings underscore the neuroprotective properties of BBR, particularly through the improvement in mitochondrial function and related processes [34].

Caffeine (Cof) is perhaps the best-known alkaloid. Min et al. showed that long-term treatment with Cof in ageing *Caenorhabditis elegans* (*C. elegans*) normalised ΔΨm and ROS generation while improving mitochondrial morphology [35]. Another study investigated the effects of Cof pretreatment on HaCaT cells (immortalised keratinocyte line) exposed to ultraviolet (UV) radiation. Cof was shown to reduce intracellular and mitochondrial ROS accumulation and inhibit the mitochondrial apoptosis pathway by downregulating the expression of Bax and cleaved caspase-3 and -9 while upregulating Bcl-2 [36].

Capsaicin (CS), known for giving chilli peppers their pungent taste, is another prominent alkaloid. Han et al. investigated the effects of CS treatment on lung cancer cell lines A549, H1299, H2009, and H23 and found that CS reduced the oxygen consumption rate (OCR), thereby decreasing ATP production in these cancer cells [37]. Furthermore, Qiao et al. investigated the effect of CS pretreatment on isolated neonatal rat cardiomyocytes exposed to lipopolysaccharide (LPS). Their results demonstrated the anti-oxidant properties of CS, as it increased the activities of superoxide dismutase (SOD), catalase (CAT), and glutathione peroxidase (GPx) while decreasing ROS and MDA levels. Notably, CS pretreatment also upregulated the expression levels of NDUFB8 (NADH dehydrogenase [ubiquinone] 1 beta subcomplex subunit 8) and UQCRC2 (cytochrome b-c1 complex subunit 2), indicating increased activities of mitochondrial complexes I and III [38].

Betanin, an alkaloid with anti-oxidant and anti-inflammatory properties, has been examined as an anticancer agent. Salimi et al. showed that betanin treatment induced mitochondria-mediated apoptosis in U87MG cells (human glioma cell line) and mitochondria isolated from them by inhibiting SDH activity, decreasing ΔΨm, and inducing extensive mitochondrial swelling, ROS generation, and CytC release. It is worth noting that betanin treatment did not affect human lymphocytes or mitochondria isolated from them [39]. Furthermore, Hafez et al. demonstrated that betanin reduced doxorubicin cardiotoxicity. Doxorubicin is an anticancer drug effective in many cancer types, but its cardiotoxicity limits its clinical applications. Researchers showed that betanin pretreatment reverted doxorubicin-induced cytotoxicity in isolated rat cardiomyocytes and mitochondria by decreasing levels of MDA and ROS generation, preventing GSH reduction, glutathione disulfide (GSSG) elevation, and mitochondrial swelling [40].

Liensinine (LIEN) is another alkaloid linked to doxorubicin cardiotoxicity. In neonatal mouse ventricular myocytes and cardiomyocytes isolated from doxorubicin-treated mice, Liang et al. demonstrated that LIEN could alleviate this dysfunction and related apoptosis by inhibiting Drp1-mediated excess mitochondrial fission. LIEN suppressed Drp1 phosphorylation at the Ser616 site—a process that promotes mitochondrial division. Moreover, LIEN treatment restored ΔΨm and inhibited ROS overproduction and CytC leakage [41]. Additionally, Wang et al. examined the influence of LIEN on colorectal cancer cell lines—HT29 and DLD-1. They showed that LIEN decreased ΔΨm and increased ROS generation. Interestingly, LIEN treatment affected apoptosis-related proteins—it upregulated the expression of Bax and downregulated the expression of Bcl-2 [42].

Matrine (MAT) is a natural alkaloid with demonstrated potential to reduce the adverse effects of chemotherapeutic agents. Cisplatin, a widely used chemotherapeutic drug for solid tumours, often accumulates in renal cells, leading to acute kidney injury and, consequently, limiting its clinical application. Yuan et al. reported that MAT can alleviate cisplatin-induced nephrotoxicity by modulating mitochondrial function. Specifically, MAT treatment reversed mitochondrial depolarisation and reduced the accumulation of reactive oxygen species (ROS) induced by cisplatin treatment in HK2 cells, a human proximal tubular epithelial cell line. Moreover, MAT excesses its anti-oxidant properties by increasing CAT activity and decreasing MDA levels. Furthermore, MAT downregulated the expression of Bax and upregulated the expression of Bcl-2 [43]. In a rat model of multiple sclerosis, Wang et al. showed that MAT in damaged oligodendrocytes was able to downregulate the expression of CytC and decrease MDA content. Moreover, MAT treatment enhanced mitophagy in those cells. Additionally, in liver cancer cell lines—HepG2 and Huh7—MAT induced oxidative stress by increasing ROS production and reducing the levels of anti-oxidant enzymes such as SOD, GSH, and GPx. Furthermore, MAT upregulated the expression of Bax, Bad, caspase-3 and -9, and cytoplasmatic CytC [44].

Huperzine A (HupA) is an alkaloid used in clinical trials as a drug for AD and other forms of dementia. Xiao et al. demonstrated that HupA decreased ROS and Aβ1–42 oligomer levels and increased ATP levels in APP/PS1 mice. Moreover, HupA attenuated CytC leakage from mitochondria [45].

Rhynchophylline (RP) is known in medicine mainly for its ability to lower blood pressure. Qin et al. examined how RP treatment affects myocardial ischemic/reperfusion-induced cardiomyocytes. RP decreased the levels of ROS and MDA, the degree of mitochondrial permeability transition pore (MPTP) opening, and the expression of cytosolic CytC, caspase-3, and caspase-9. On the contrary, RP increased the activities of SOD and GPx [46]. Additionally, Liu et al. showed that in cardiomyocytes isolated from a mouse model of diabetic cardiomyopathy, RP treatment downregulated the expression levels of caspase-3 and Bax and upregulated the expression of Bcl-2 protein. Moreover, mitochondria were swelled before RP intervention, and ATP content was low. After RP treatment, mitochondrial morphology improved, and ATP levels increased [47]. Taken together, RP treatment alleviated mitochondria-related disruptions in injured cardiomyocytes.

Piperine (PIP) is another alkaloid whose effects were investigated by Guo et al. in HGC-27 cells (undifferentiated human gastric cancer cell line). Their results showed that PIP treatment led to increased ROS generation, increased expression levels of Bax, CytC, and activated caspase-3 and -9, accompanied by a decrease in ΔΨm [48]. Conversely, in an animal model of ischaemic stroke, Kaushik et al. demonstrated that pretreatment with PIP ameliorated post-injury oxidative stress by reducing ROS generation, restoring ΔΨm, decreasing LPO, and increasing GSH levels. In addition, they reported that PIP prevented disruptions in the activity levels of mitochondrial complexes I and II and inhibited the mitochondrial apoptosis pathway by downregulating the expression of mitochondrial Bax [49].

### 3.2. Terpenoids

**Figure 5:**
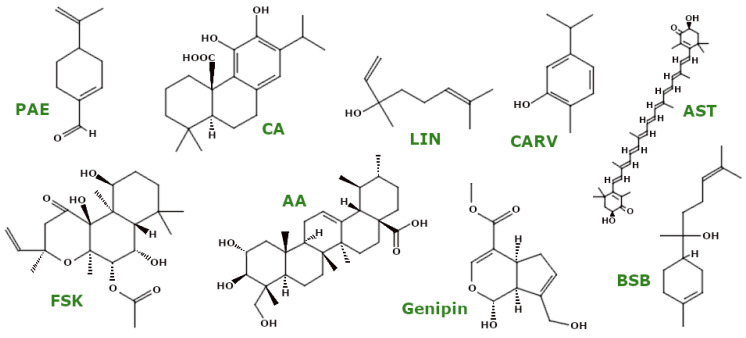
Chemical structures of perillaldehyde (PAE), asiatic acid (AA), α-bisabolol (BSB), astaxanthin (AST), forskolin (FSK), carvacrol (CARV), carnosic acid (CA), linalool (LIN), and genipin.

Terpenoids (Figure 5) are a diverse class of biologically active compounds in plants, characterised by five-carbon isoprene units that form the basis for further classification [50]. Similar to other PSMs, these molecules also influence mitochondrial parameters.

Perillaldehyde (PAE), a monoterpene with known anti-oxidant properties, has been investigated for its effects on mitochondrial health. Fang et al. showed that in a *C. elegans* model of HD, PAE increased ATP levels and activated the mitochondrial unfolded protein response (UPRmt), a critical process for maintaining mitochondrial integrity and promoting cell survival [51].

Asiatic acid (AA), a triterpene, was studied by Varada et al. in both healthy and 5xFAD mice, the latter being an animal model of AD due to the overexpression of APP and PS1 proteins with five mutations associated with familial AD. Their results showed that AA increased basal mitochondrial respiration, probably by upregulating the genes encoding ETC complexes. In addition, AA increased the expression of the anti-oxidant genes *NRF2*, *HMOX1*, and *GCLC*, thereby protecting ETC-encoding genes [52]. In a model of myocardial I/R injury, AA pretreatment increased SOD activity, decreased MDA levels, reduced ROS production, and stabilised ΔΨm, thereby preserving mitochondrial morphology [53]. In a rat model of epilepsy, AA also affected mitochondrial proteins, including the mitochondrial deacetylase sirtuin-3 (SIRT3) and ATP synthase (ATP5A), suggesting improved mitochondrial function [54].

α-Bisabolol (BSB) (levomenol), an unsaturated sesquiterpene alcohol, has shown potential in reducing oxidative stress. Javed et al. found that in rotenone-treated rats, BSB increased anti-oxidant enzyme levels (e.g., SOD, CAT) and reduced lipid peroxidation (MDA) while also increasing GSH levels. In addition, BSB inhibited mitochondrial apoptosis by increasing Bcl-2 expression and decreasing Bax and cleaved caspase-3 and -9 levels, thereby reducing mitochondrial membrane permeabilisation and cell death [55].

Astaxanthin (AST), a xanthophyll, has been shown to have protective effects on mitochondria in SH-SY5Y human neuroblastoma cells exposed to H_2_O_2_. Brasil et al. reported that pretreatment with AST reduced CytC release and the levels of pro-apoptotic caspase-3 and -9. It also protected mitochondrial membranes by reducing protein carbonylation and nitration, increasing ΔΨm, and increasing the activities of mitochondrial complexes I and V, resulting in increased ATP levels [56]. In addition, AST reduced mitophagy in a similar model by activating the Akt/mTOR pathway [57].

Forskolin (FSK), a diterpenoid [58], has shown significant anti-oxidant effects in a PD model using 6-hydroxydopamine (6-OHDA)-treated rats. Alharbi et al. found that FSK increased the activity of mitochondrial complexes (I, II, and V) while increasing SOD, CAT, and GSH levels and decreasing MDA and H_2_O_2_ levels, highlighting its anti-oxidant activity [59].

Carvacrol (CARV), a monoterpene, showed anti-oxidant properties in lead toxicity models. Zare Mehrjerdi et al. showed that CARV increased CAT levels and SOD activity in lead-exposed rats [60]. In addition, CARV stabilised ΔΨm and prevented mitochondrial ROS elevation in SH-SY5Y, BV-2 (murine microglia), and HEK293 (human embryonic kidney) cells [61].

Carnosic acid (CA) is a potent anti-oxidant. In a mouse model of chlorpyrifos (CPF)-induced neurotoxicity, AlKahtane et al. showed that CA pretreatment reduced MDA and NO levels while increasing SOD, CAT, GSH, and GPx levels [62]. In addition, Lin et al. found that CA modulated mitochondrial dynamics in 6-OHDA-treated SH-SY5Y cells by increasing the expression of the fusion proteins Opa1 and Mfn2 and decreasing the expression of the fission proteins Fis1 and Drp1, thereby supporting mitochondrial fusion. CA also prevented mitochondrial CytC release [63]. Another study by the same group showed that CA pretreatment increased PGC-1α protein levels and upregulated the mRNA levels of genes related to mitochondrial biogenesis [64].

Linalool (LIN), an acyclic monoterpene, has shown anti-oxidant effects in isolated human lymphocytes exposed to benzene. Salimi et al. reported that LIN reduced mitochondrial ROS production, decreased MDA levels, stabilised GSH, and regulated ΔΨm [65].

Genipin, an iridoid compound from the leaf extract of Corema album, showed bioactivity against PD. Rosado-Ramos et al. found that in genetically modified yeast cells overexpressing α-synuclein-GFP, genipin downregulated the genes encoding mitochondrial complex IV proteins (e.g., COX4 and COX13), thereby modulating OXPHOS, a major source of ROS [66]. In models of diabetic retinopathy, Sun et al. showed that genipin reduced oxidative stress in human retinal microvascular endothelial cells exposed to high glucose and in streptozotocin-treated mice, stabilising ROS generation, ATP levels, and ΔΨm [67].

### 3.3. Saponins

**Figure 6:**
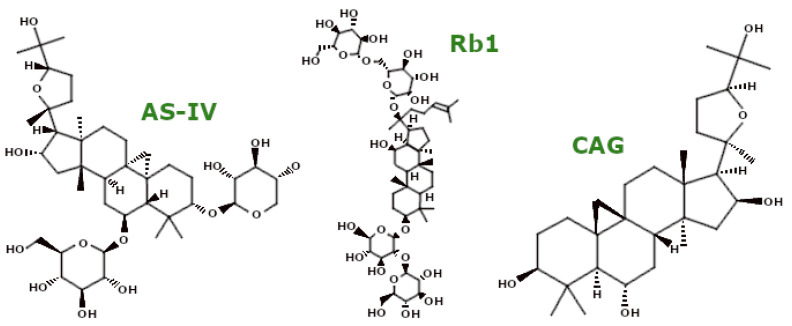
Chemical structures of Ginsenoside Rb1 (Rb1), astragaloside IV (AS-IV), and cycloastragenol (CAG).

Saponins (Figure 6) are naturally occurring low-surfactant emulsifiers characterised by a triterpenoid or steroid backbone attached to several saccharide residues [68]. In particular, Platycodon grandiflorum contains about 20 active saponins, with platycodin D and platycoside E as the major components. Ji et al. showed that pretreatment with Platycodon grandiflorum crude saponin (PGS) in HT22 mouse hippocampal cells reduced ROS production. Furthermore, PGS activated the Nrf2 pathway and upregulated genes for anti-oxidant enzymes such as haem oxygenase-1 (HO-1), SOD, and CAT. PGS also inhibited mitochondrial apoptosis by suppressing Bax expression and increasing Bcl-2 levels, preventing CytC release and thus averting activation of caspase-3 and -9 [69].

Panax japonicus (SPJ) represents another plant rich in saponins. Its saponin compounds were studied by Fan et al. in both ageing rats and D-galactose-treated SH-SY5Y cells. In both models, SPJ improved mitochondrial morphology and dynamics by decreasing Drp1 protein levels and increasing the mitochondrial fusion proteins Mfn2 and Opa1. In cellular models, SPJ also downregulated ROS production and increased ∆Ψm and ATP levels [70].

Marsdenia tenacissima saponins (SMT) were investigated by Jiang et al. for their mitochondrial effects in four liver cell lines: two human hepatocellular carcinoma lines (HepG2 and Li-7), one normal human liver cell line (L-02), and one murine hepatocellular carcinoma line (H22). SMT induced mitochondrial swelling and cristae fragmentation, impaired mitochondrial function, and activated mitochondrial apoptosis. SMT upregulated Bax, CytC, and caspase-9 and cleaved caspase-3 expression, facilitating cell death [71].

Two recently identified steroidal saponins, deoxytrillenoside CA (DTCA) and epitrillenoside CA (ETCA), were evaluated by Wu et al. in a *C. elegans* model of Huntington’s disease. Their study revealed that both compounds enhanced mitochondrial function through multiple mechanisms: they reduced ROS generation, increased mitochondrial content, and upregulated respiratory chain subunit genes including *CCO-1*, *MEV-1*, *MRPS-5*, *NDUT-6*, *PHI-37*, and *SDHA-1*, thus stimulating mitochondrial biogenesis. These saponins also upregulated genes such as *PINK1*, *DCT-1*, and *PDR-1*, thereby enhancing mitophagy [72].

Ginsenoside Rb1 (Rb1) is another saponin of note. In a rat model of brain ischaemia and primary astrocytes, Ni et al. reported that Rb1 decreased mitochondrial ROS production and increased GSH levels, showing anti-oxidant activity. In addition, Rb1 reversibly inhibited mitochondrial complex I activity [73]. Jiang et al. further demonstrated the anti-oxidant potential of Rb1 in a mouse I/R injury model and isolated cardiomyocytes, where it inhibited mitochondrial ROS generation, stabilised mitochondrial permeability transition pore (mPTP) activity, and maintained ∆Ψm. Rb1 pretreatment also downregulated the pro-apoptotic proteins Bax and Bak and upregulated the anti-apoptotic Bcl-2 [74].

Astragaloside IV (AS-IV), a saponin known for its anti-oxidant properties, was studied by Xia et al. in an MPTP-induced mouse model of Parkinson’s disease. They demonstrated that AS-IV pretreatment enhanced mitochondrial function through two mechanisms: reducing ROS production and stimulating mitophagy via the upregulation of PINK1 and Parkin expression [75]. Zhang et al. further investigated the effects of AS-IV in a myasthenia gravis rat model and showed that AS-IV improved mitochondrial ultrastructure, increased ATPase activity, and promoted mitophagy by increasing PINK1, Parkin, and LC3 II expression. In addition, AS-IV inhibited mitochondrial apoptosis by downregulating CytC, Bax, caspase-3, and caspase-9 and upregulating Bcl-2 [76]. In a mouse model of diabetic nephropathy and mouse podocyte cells (MPCs), Li et al. reported that AS-IV reduced ROS levels, increased the activity of key anti-oxidant enzymes (CAT, SOD2, HO-1), and enhanced GPx activity while improving mitochondrial morphology and promoting mitochondrial biogenesis via the SIRT1/PGC-1α/Nrf1 pathway [77]. Luo et al. further demonstrated the benefits of AS-IV in aged rats, where it increased basal and maximal mitochondrial respiration rates and ATP production by enhancing mitochondrial complex I-IV activity [78]. In a rat model of diabetic peripheral neuropathy, Ben et al. showed that AS-IV improved mitochondrial morphology, increased mitochondrial complex (I–IV) activity, and increased ∆Ψm while decreasing MDA and increasing GSH levels [79].

Cycloastragenol (CAG), the active form of AS-IV, shows remarkable neuroprotective effects. Ikram et al. showed that in an AD mouse model (induced by Aβ1–42 peptide injection), CAG reduced oxidative stress by decreasing ROS and LPO levels and inhibited mitochondrial apoptosis by increasing Bcl-2 and decreasing Bax, caspase-3, and Bim levels [80]. In a middle cerebral artery occlusion model, Li et al. reported that CAG treatment reduced the Bax/Bcl-2 ratio, further supporting its neuroprotective potential [81].

### 3.4. Polyphenols

Based on their phenolic ring structures and functional groups, polyphenols encompass four main categories of phytochemicals: phenolic acids, flavonoids, stilbenes, and lignans [82].

#### 3.4.1. Polyphenols—EVOO Polyphenols

**Figure 7:**
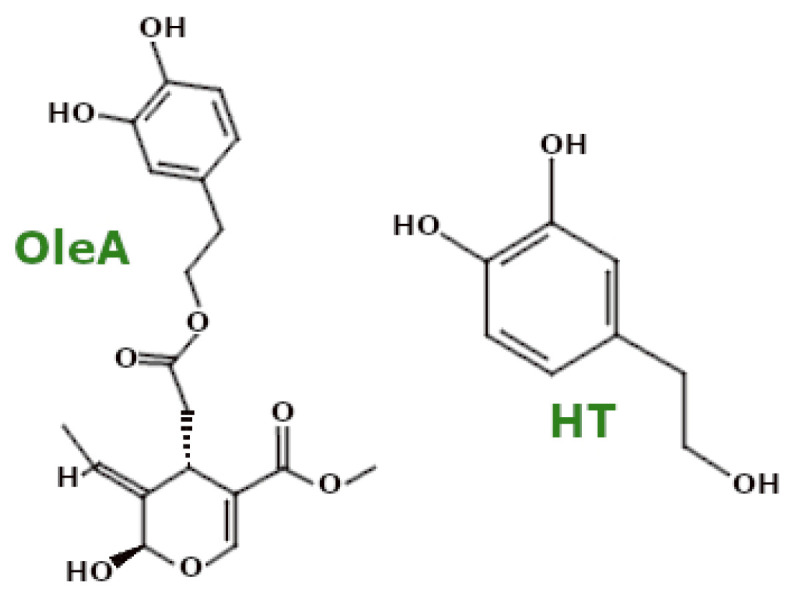
Chemical structures of oleuropein aglycone (OleA) and hydroxytyrosol (HT).

Extra virgin olive oil (EVOO) is particularly rich in polyphenols, especially oleuropein aglycone (OleA) and its metabolite hydroxytyrosol (HT) (Figure 7). Pretreatment with a mixture of these compounds in SH-SY5Y cells exposed to Aβ_1-42_ oligomers resulted in reduced ROS levels, demonstrating their anti-oxidant properties [83].

#### 3.4.2. Polyphenols—Flavonoids

**Figure 8:**
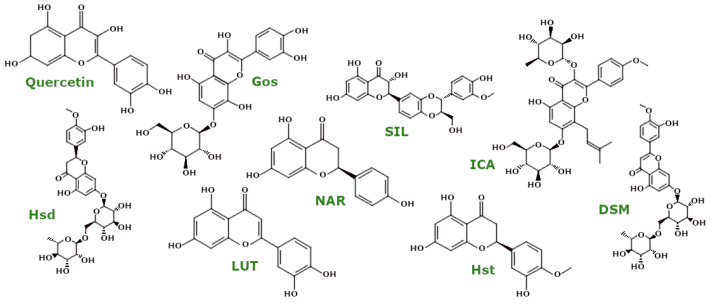
Chemical structures of quercetin, naringenin (NAR), gossypitrin (Gos), silibinin (Silybin, SIL), luteolin (LUT), hesperetin (Hst), hesperidin (Hsd), diosmin (DSM), and icariin (ICA).

Quercetin (Figure 8), a well-known flavonoid, has shown efficacy in reducing oxidative stress. In RTN-treated rats, quercetin reduced MDA levels, increased GSH levels, and increased SOD activity. Its effects were more pronounced with pretreatment, supporting the importance of prevention in ND models [84]. Quercetin also restored mitochondrial integrity and reduced ROS in PC12 cells treated with 6-OHDA, increased SOD activity, and reduced MDA and ROS levels in the substantia nigra of 6-OHDA-treated rats while upregulating PINK1 and Parkin proteins [85]. In studies using a cellular model of alcoholic liver disease (ethanol-treated L02 cells), quercetin reduced CYP2E1 activity and subsequently attenuated ROS and mitochondrial dysfunction. This compound also affected mitochondrial dynamics by upregulating Opa1 and Mfn1 and downregulating Mff, Drp1, and Fis1 [86].

Naringenin (NAR) (Figure 8), a major flavanone, showed neuroprotective effects in 6-OHDA-treated SH-SY5Y cells by reducing ROS levels and normalising CAT, GSH, and SOD levels with an associated increase in ΔΨm. In *Danio rerio* models treated with 6-OHDA, NAR downregulated *LRRK2* and *POLG* while upregulating *PINK1*, genes implicated in PD [87]. In PQ-treated SH-SY5Y cells and rat models, NAR reduced ROS levels, increased ΔΨm and ATP levels, and promoted the mitochondrial apoptosis pathway through caspase activation and modulation of Bcl-2 and Bax expression [88,89,90].

Fisetin, apigenin, and chrysin are flavonoids that have shown potential in mitochondrial uncoupling and ΔΨm depolarisation, thereby attenuating neurodegeneration in *C. elegans* models [91]. Fisetin and sterubin also reduced mitochondrial ROS and Ca^2+^ overload while maintaining the activity of the AMPK/SIRT1/PGC-1α pathway, which is critical for mitochondrial biogenesis under ferroptosis-related stress conditions [92].

Gossypitrin (Gos) (Figure 8) has been tested in HT22 cells and rat forebrain mitochondria. Gos reduced mitochondrial LPO, decreased swelling, and increased both ΔΨm and ATP levels [93].

Silibinin (Silybin, SIL) (Figure 8) is a flavonolignan and the primary active compound in silymarin, a well-known hepatoprotective agent. In an animal model of PD using MPTP-treated mice, Liu et al. demonstrated that SIL alleviates oxidative stress by reducing ROS and MDA levels while increasing GPx activity. Additionally, SIL enhanced PINK1 and Parkin expression, increasing the LC3 II/I ratio and indicating mitophagy activation and clearance of damaged mitochondria [94]. Further studies showed that SIL improved redox balance by enhancing SOD activity, decreasing MDA levels, and exerting anti-apoptotic effects by elevating Bcl-2 levels while decreasing Bax, cleaved caspase-3, and caspase-9 expression. SIL also modulated mitochondrial dynamics by decreasing Drp1 expression and increasing Mfn1 expression, resulting in improved mitochondrial length and structure [95]. Tie et al. reported similar outcomes in SH-SY5Y cells treated with H_2_O_2_ [96].

In a cellular model of early AD using PC12APPsw cells, Esselun et al. observed that SIL reduced ROS production and improved ATP levels, ΔΨm, and membrane fluidity. SIL also inhibited mitochondrial rupture by reducing Ca^2^^+^-induced mitochondrial swelling [97]. In another study, Iyengar and Devaraj found that SIL induced apoptosis in SCC-25 cells (human oral squamous carcinoma cell line) by upregulating the expression of pro-apoptotic proteins Bax, CytC, caspase-3, and caspase-9 while downregulating anti-apoptotic Bcl-2 expression [98].

Luteolin (LUT) (Figure 8), a flavone, has shown promising effects on mitochondrial health. Naia et al. reported that LUT treatment enhanced respiration and ATP production in differentiated SH-SY5Y cells without affecting mitochondrial structure or inducing mitochondrial ROS accumulation. Interestingly, LUT increased the number of mitochondria–endoplasmic reticulum (ER) contacts, contributing to higher mitochondrial Ca^2^^+^ levels and enhancing the activity of mitochondrial complexes I and II [99]. LUT also shows cardioprotective effects. Xu et al. demonstrated that it reduced apoptosis and ROS accumulation while increasing ΔΨm. Additionally, LUT promoted mitochondrial autophagy by enhancing Drp1 phosphorylation at Ser616 [100]. In gastric cancer cell lines (HGC-27, MFC, MKN-45), LUT increased ROS levels while decreasing ΔΨm, ATP content, and activity levels of SOD, Na^+^/K^+^-ATPase, and Ca^2^^+^/Mg^2^^+^-ATPase. LUT downregulated Bcl-2 and upregulated Bax expression, contributing to apoptosis [101]. Chen et al. found that LUT increased ATP content and ATPase activity in isolated chicken spleen lymphocytes treated with ammonium chloride, downregulating Drp1, Opa1, and Mff mRNA expression [102].

Flavonoid-rich plant extracts and their isolated compounds have been extensively studied for their effects on mitochondrial function and biogenesis. Mandarin juice flavonoid-rich extract (MJe), primarily containing hesperidin, demonstrated anti-oxidant effects in 6-OHDA-treated SH-SY5Y cells by reducing ROS accumulation and increasing the activity of SOD, CAT, and GSH, along with elevating ΔΨm. MJe also regulated PD-related gene expression, reducing *PINK1* and *PARK2* and thus promoting mitochondrial survival. Additionally, MJe decreased Bax and p53 levels while increasing Bcl-2, which collectively inhibited mitochondrial apoptosis pathways [103]. Hesperidin (Hsd), a flavonoid glycoside, improved mitochondrial integrity in 6-OHDA-treated mice by increasing the activity of mitochondrial complexes I, IV, and V, as well as Na^+^/K^+^-ATPase, along with elevating ΔΨm. Hsd also attenuated caspase-3 and caspase-9 activity, revealing its anti-apoptotic potential [104].

Hesperetin (Hst) (Figure 8), a flavonoid with greater bioavailability than Hsd, has also been shown to modulate mitochondrial function. Lv et al. observed that Hst increased ROS levels and inhibited GPx4 expression in bladder cancer cell lines (T24[HTB-4] and 5637[HTB-9]). Additionally, Hst upregulated Bax and cleaved caspase-3 expression while downregulating Bcl-2, leading to decreased mitochondrial volume and increased membrane density [105]. In HepG2 cells treated with palmitic acid to model NAFLD, Li et al. demonstrated that Hst elevated maximal respiration, spare respiratory capacity, and OCR linked to ATP production. Hst also increased ΔΨm and suppressed mitochondrial ROS production, activating PINK1/Parkin-mediated mitophagy [106].

Diosmin (DSM) (Figure 8), a flavonoid glycoside derived from hesperidin, improved mitochondrial complex I and II activity in quinolinic acid-treated rats, a model of neurodegeneration, and increased endogenous anti-oxidant activity [107].

Icariin (ICA) (Figure 8), like Hsd, is a flavonoid glycoside that modulates mitochondrial function. In PC12 cells exposed to D-galactose, Hu et al. found that ICA treatment reduced excessive autophagy by lowering the LC3B II/I ratio and increasing p62 expression. ICA also reduced ROS production, enhanced ΔΨm, and mitigated mPTP opening under stress conditions, which reversed autophagy activation [108]. Additionally, in nucleus pulposus (NP) cells treated with H_2_O_2_ to mimic intervertebral disc degeneration (IDD), ICA pretreatment attenuated ROS and CytC release, improved mitochondrial morphology, and enhanced ΔΨm. ICA further inhibited mitochondrial apoptosis by upregulating Bcl-2 while downregulating Bax and cleaved caspase-3 expression [109]. In cancer cell lines such as MDA-MB-231, MDA-MB-453, and 4T1 (mouse mammary carcinoma cell line), ICA promoted mitochondrial apoptosis by upregulating cleaved caspase-3 and Bax while downregulating Bcl-2, which led to a loss in ΔΨm and increased ROS [110]. Finally, Wang et al. observed that combined treatment with β-asarone and ICA enhanced PINK1/Parkin-mediated mitophagy in Aβ_1-42_-treated PC12 cells and APP/PS1 mice [111].

Catechins, a group of polyphenols including epigallocatechin gallate (EGCG), gallocatechin gallate (GCG), gallocatechin (GC), epicatechin gallate (ECG), and epicatechin (EC), have potent anti-oxidant properties. Park et al. reported that these catechins exhibited free radical scavenging effects in HT22 cells under oxidative stress induced by glutamate, with GCG, in particular, reducing ROS levels by inhibiting intracellular Ca^2^^+^ influx and accumulation [112].

#### 3.4.3. Polyphenols—Non-Flavonoid Polyphenols

Among polyphenols, the non-flavonoid subclass includes stilbenes, lignans, tannins, and their derivatives. A prominent example is the lignan magnolol (MGN), which has been investigated for its anti-oxidant properties. Yu et al. demonstrated the anti-oxidant activity of MGN in ageing *C. elegans*, showing reduced MDA levels and increased SOD and CAT enzyme activities. In addition, MGN treatment was found to protect mitochondrial morphology [113].

**Figure 9:**
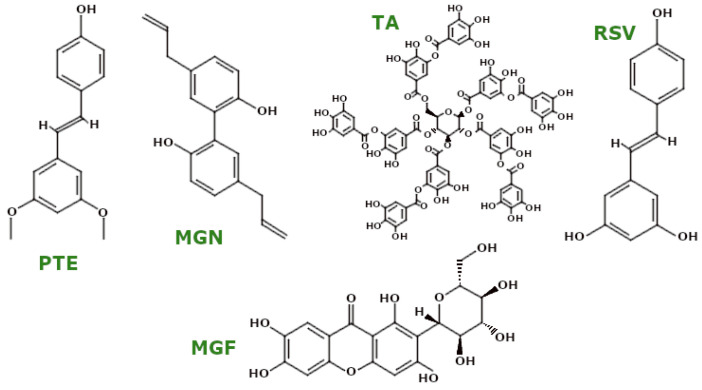
Chemical structures of magnolol (MGN), tannic acid (TA), resveratrol (RSV), pterostilbene (PTE), and mangiferin (MGF).

Magnolol (MGN) (Figure 9) enhanced mitochondrial function in human chondrocytes isolated from osteoarthritis patients, as reported by Liu et al. Specifically, MGN treatment increased mitochondrial membrane potential (ΔΨm), mitochondrial DNA copy number, mitochondrial mass, and ATP production. It also reduced the generation of ROS and increased SOD activity, effects attributed to activation of the SIRT1/AMPK/PGC-1α pathway [114].

To enhance the therapeutic potential of phytochemicals, researchers have developed semi-synthetic derivatives. For instance, Cheng et al. synthesised mito-magnolol (mito-MGN), a mitochondria-targeted derivative of magnolol, and evaluated its effects in melanoma cell lines. Mito-MGN increased ROS generation and induced mitophagy, inhibited mitochondrial complex I, and decreased ΔΨm, contributing to increased cancer cell death [115]. Another approach involves hybrid compounds. Tao et al. developed a magnolol-sulforaphane hybrid, CT1-3, which showed pro-apoptotic effects in cancer cells by increasing ROS generation and Bax expression while decreasing ΔΨm and Bcl-2 levels [116].

Tannic acid (TA) (Figure 9), a representative tannin, has also shown promising anti-oxidant effects. Azimullah et al. showed that TA reduced MDA and NO levels while increasing the activity of CAT, SOD, and GSH in RTN-treated rats. TA pretreatment attenuated the mitochondrial apoptotic pathway by upregulating Bcl-2 and downregulating Bax and CytC levels [117]. In addition, Salman et al. reported that TA treatment in a rodent model of traumatic brain injury modulated GSH levels, reduced LPO, and increased the activity of glutathione S-transferase (GST), GPx, CAT, and SOD. TA also upregulated the expression of PGC-1α and Nrf2 and acted as an anti-apoptotic agent [118]. In HTB-9 cells, a human bladder cancer cell line, Li et al. found that TA activated mitochondrial apoptosis through the upregulation of Bax and Bak and the downregulation of Bcl-2, while CytC expression was decreased [119].

Resveratrol (RSV) (Figure 9) is perhaps the most widely recognised polyphenol, often noted for its presence in red wine. Zeng et al. found that RSV pretreatment protected mitochondrial morphology in septic rats induced by caecal ligation and puncture, as well as in H9c2 rat cardiomyocytes, increasing GPx4 levels and ΔΨm and restoring oxidative balance [120]. Furthermore, in neonatal rat cardiomyocytes exposed to hypoxia/reoxygenation, RSV increased ΔΨm and SOD activity while decreasing MDA levels. RSV also increased mitochondrial number and function and promoted mitophagy by increasing Parkin and LC3 II expression [121]. A study by Wu et al. investigated the role of RSV in LPS-induced liver injury in *Carassius gibelio*, showing decreased MDA, LPO, and ROS levels, while NRF2 and HO-1 expression were upregulated. RSV also modulated mitochondrial dynamics by downregulating DRP1, FIS1, and MFF and upregulating MFN1, MFN2, NRF1, and TFAM, thereby activating SIRT1 and PGC-1α [122].

RSV has also been shown to improve mitochondrial biogenesis in several models. For example, in SH-SY5Y cells exposed to fluoride toxicity, RSV pretreatment upregulated SIRT1 deacetylase activity without altering SIRT1 protein expression, activating the SIRT1-dependent PGC-1α/NRF1/TFAM pathway, which induced mitochondrial biogenesis. In fluoride-treated rats, RSV treatment improved mitochondrial morphology and mtDNA content, supporting mitochondrial health in hippocampal neurons [123]. Adedara et al. studied RSV in a *Drosophila melanogaster* model of PD. They found reductions in H_2_O_2_, MDA, and NO levels, with increases in non-protein and total thiols. In addition, RSV upregulated CAT and GST activities, increased mitochondrial mass, and upregulated *PLE* and *SOD1* genes [124]. In colorectal cancer cell lines HCT116 and SW620, Fu et al. reported that RSV increased ROS production and induced apoptosis by downregulating Bcl-2 and upregulating Bax, CytC, and cleaved caspase-3 and -9 [125].

Pterostilbene (PTE) (Figure 9), a natural methoxylated analogue of RSV, has shown higher bioavailability. Yan et al. showed that pretreatment with PTE significantly increased endogenous anti-oxidant enzymes (SOD, CAT, GPx, GSH) and reduced MDA levels in a rat model of cerebral ischaemia [126]. Wu et al. further reported that PTE treatment in a mouse model of intracerebral haemorrhage (ICH) exhibited anti-apoptotic properties by increasing Bcl-2 and decreasing Bax. PTE treatment in BV2 cells, a model of ICH, improved mitochondrial morphology and ΔΨm and reduced mitochondrial ROS. In addition, PTE upregulated Opa1 expression via Nrf2, suggesting that the regulatory effects of PTE on mitochondrial morphology occur in an Nrf2-dependent manner [127]. In turn, Gao et al. demonstrated the effects of PTE in human and rat glioma cell lines, showing that PTE induced apoptosis by increasing Bax and cleaved caspase-3 and -9 and decreasing Bcl-2 levels. PTE also disrupted ΔΨm, increased ROS, and decreased GSH levels [128].

Mangiferin (MGF) (Figure 9), a xanthonoid polyphenol, has shown significant mitochondrial protective effects. Tang et al. showed that MGF pretreatment prevented mitochondrial apoptosis and modulated mitochondrial dynamics in fluoride-exposed SH-SY5Y cells, with an increase in Bcl-2 and a decrease in caspase-3 and -9 and Drp1 [129]. In human retinal pigment epithelial cells (ARPE-19) treated with H_2_O_2_, Park et al. showed that MGF increased ΔΨm, decreased CytC release, increased MnSOD and GPx activities, and decreased mitochondrial ROS production [130]. In MPTP-treated mice, MGF improved mitochondrial morphology and ATP levels while inhibiting mitochondrial Drp1 translocation to prevent excessive fission. MGF also upregulated PINK1 and Parkin and promoted mitophagy [131]. In models of IDD, MGF improved mitochondrial morphology and function in human peripheral nerve cells by increasing ΔΨm, reducing ROS, and modulating apoptosis-related mRNA expression [132].

#### 3.4.4. Polyphenols—Ellagitannins

**Figure 10:**
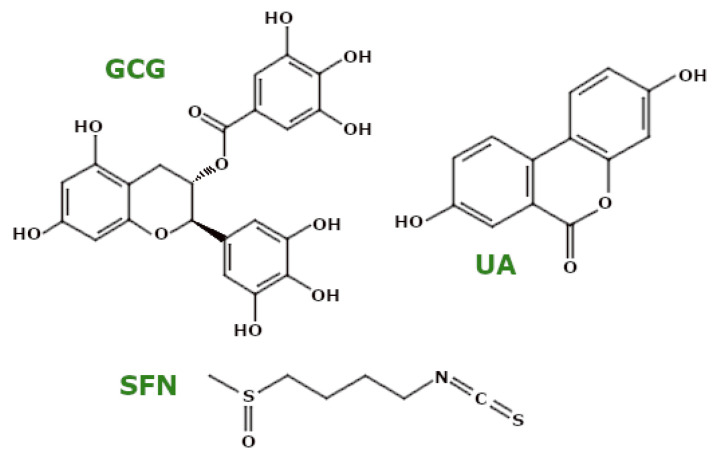
Chemical structures of gallocatechin gallate (GCG), urolithin A (UA), and sulforaphane (SFN).

Urolithin A (UA) (Figure 10), a metabolite generated by gut microbiota from ellagitannins, is a notable phytochemical with diverse mitochondrial effects. Research has shown that UA treatment upregulates key mitochondrial genes, including *ATP5D* (encoding complex V), *NDUFV1* (encoding complex I), and *CS* (a mitochondrial marker), thereby enhancing OXPHOS and mitochondrial biogenesis in SY5Y-APP695 cells, a cellular model of early AD [133]. Cho et al. demonstrated that in H_2_O_2_-induced senescent House Ear Institute-Organ of Corti 1 cells, as well as cochlear explants from mice, UA pretreatment increased mRNA levels of the genes associated with mitophagy, such as *PINK1*, *PARK2*, and *BNIP3*, while reducing senescence markers like *p21*. Additionally, UA downregulated *p53* and increased protein levels of PINK1, Parkin, and BNIP3, suggesting an induction of mitophagy. Furthermore, UA was observed to reduce ΔΨm depolarisation, thereby restoring it to normal levels [134].

In human primary chondrocytes isolated from osteoarthritic knee cartilage, UA treatment was reported by D’Amico et al. to enhance basal, maximal, and ATP-linked mitochondrial respiration while also increasing mitophagy via the PINK1/Parkin-mediated pathway [135]. Similarly, Singh et al. showed that UA treatment in isolated human skeletal muscle cells activated the PINK1/Parkin pathway, increased phospho-Parkin (Ser65) levels, and facilitated Parkin translocation to mitochondria during mitophagy. UA also elevated protein levels of mitochondrial complexes I, II, and III, suggesting an overall enhancement in mitochondrial function [136]. Furthermore, Kshirsagar et al. investigated UA in combination with EGCG in humanised β-amyloid knockin (hAbKI) mice, a model of late-onset AD, finding that this treatment improved mitochondrial ATP levels, reduced lipid peroxidation and free radical levels, and decreased mitochondrial fragmentation. Notably, the combination treatment increased mitochondrial length and facilitated the removal of damaged mitochondria through increased autophagosome formation [137].

### 3.5. Glucosinolates

Sulforaphane (SFN) (Figure 10), an isothiocyanate derived from glucosinolates, has shown protective effects on mitochondrial function [138]. Brasil et al. reported that in CPF-treated SH-SY5Y cells and LPS-exposed mouse BV2 cells, SFN pretreatment suppressed CytC release and reduced ROS and nitric oxide synthase (NOS) production. Additionally, SFN lowered the levels of mitochondrial membrane markers such as MDA, 3-nitrotyrosine (a protein nitration marker), and protein carbonylation (indicative of oxidative protein damage). SFN enhanced ΔΨm and increased the activity of mitochondrial complexes I, II, III, and V, thereby boosting ATP production [139]. Folbergrová et al. also observed a reduction in 3-nitrotyrosine levels in a rodent model of epilepsy treated with SFN [140]. In another study, Napoli et al. showed that in fibroblasts derived from fragile X-associated tremor/ataxia syndrome patients, SFN decreased mitochondrial ROS and proton leakage, thereby enhancing coupling efficiency [141].

### 3.6. Ethanolic Extract of Mentha Peperita (EthMP)

Researchers also study whole plant extracts to investigate how their combined secondary metabolites affect mitochondrial function. Anjum et al. studied the ethanolic extract of Mentha peperita (EthMP) and its anti-oxidant properties in mice treated with RTN. The study showed that EthMP reduced LPO levels and enhanced SOD and CAT activities, indicating its antioxidative potential [142].

### 3.7. Phytocannabinoids

**Figure 11:**
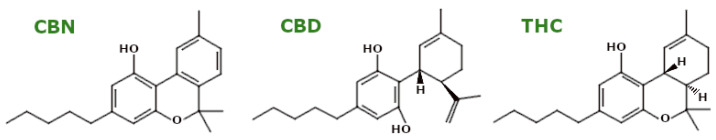
Chemical structures of cannabinol (CBN), Δ9-tetrahydrocannabinol (THC), and cannabidiol (CBD).

Cannabinoids (Figure 11), including cannabinol (CBN), Δ9-tetrahydrocannabinol (THC), and cannabidiol (CBD), have received increasing attention for their diverse effects on mitochondrial function and dynamics. CBN has been shown to modulate a variety of mitochondrial mechanisms. Liang et al. reported that pretreatment with CBN in glutamate-treated HT22 cells induced anti-oxidant effects, including reduced production of ROS and LPO and the upregulation of superoxide dismutase 2 (SOD2) and glutathione peroxidase 4 (GPx4) [143]. In addition, CBN suppressed mitochondrial heat shock protein 60 (HSP60), a stress-induced protein, and prevented mitochondrial calcium overload by regulating the mitochondrial calcium uniporter (MCU). CBN also affected OXPHOS by modulating the activities of mitochondrial complexes I, III, IV, and V and improved mitochondrial dynamics by upregulating Opa1 and Mfn2, thereby promoting mitochondrial fusion and increasing mitochondrial mass [143].

Phytocannabinoids such as THC and CBD also exert significant effects on mitochondrial function, particularly in relation to the activities of the ETC complex and endocannabinoid system (ECS). Noskova et al. highlighted that THC and CBD modulate mitochondrial respiratory chain activity, resulting in changes in ROS production and cellular integrity [144,145]. Depending on concentration and context, these cannabinoids can have both protective and deleterious effects on mitochondrial health. For example, THC has been shown to disrupt mitochondrial respiration at low concentrations, inducing mitochondrial depolarisation and altered bioenergetics in transformed lung cells and brain mitochondria [144].

The ECS is a complex cell-signalling network that plays a crucial role in maintaining physiological homeostasis. It consists primarily of endogenous ligands (endocannabinoids), cannabinoid receptors, and enzymes responsible for their synthesis and degradation. Cannabinoid receptor-mediated pathways, primarily through CB1 and CB2 receptors, play a central role in mitochondrial modulation. Yang et al. demonstrated that CB1 receptor activation by arachidonyl-2-chloroethylamide (ACEA), a selective agonist, attenuated brain ischaemia/reperfusion injury by restoring mitochondrial function and possibly inhibiting MPTP opening [146]. Further evidence from Mendizabal-Zubiaga et al. indicated that CB1 receptors localised in striated muscle cells regulate mitochondrial respiration, suggesting a direct link between receptor activation and mitochondrial bioenergetics [147].

Preclinical studies have further highlighted the influence of cannabinoids on mitochondrial dynamics in various models. Pinky et al. observed stage-specific effects of cannabinoid exposure on mitochondrial function, with prenatal exposure in rat offspring increasing complex I and complex IV activities, in contrast to findings in adult models [148]. Ma et al. also showed that CB1 receptor activation improves mitochondrial function in neurons, highlighting the protective role of cannabinoid signalling in maintaining mitochondrial integrity [149]. In cancer research, Rupprecht et al. reported that combined THC and CBD treatment suppressed mitochondrial respiration in glioblastoma cells through the downregulation of specific respiratory chain proteins. These findings highlight the potential of cannabinoids to modulate mitochondrial function in oncological contexts and offer promising avenues for therapeutic strategies targeting mitochondrial health in malignancies [150].

## 4. Future Perspective in Mitochondria Modulation: Synthetic, Semi-Synthetic, and PSMs in Drug Development

Modulation of mitochondrial health is a critical area of research, particularly in the context of drug development. This field has given rise to synthetic and semi-synthetic drugs, as well as plant secondary metabolites, each with unique mechanisms of action and therapeutic benefits. A comprehensive comparison of these categories highlights their potential to synergistically improve mitochondrial function and overall cellular health.

Synthetic drugs such as hexestrol have been recognised for their ability to modulate mitochondrial dynamics. Delerue et al. demonstrated that hexestrol targets Dnm1p-dependent mitochondrial fission, suggesting its potential utility in the treatment of mitochondrial disorders by affecting mitochondrial morphology and function [151]. Similarly, compounds such as mdivi-1, which directly inhibits DRP1, highlight the ability of synthetic drugs to target mitochondrial dysfunction [151]. Another example is ZLN005, a PGC-1α agonist, which promotes the upregulation of PGC-1α activity and enhances mitochondrial biogenesis. Both compounds are derived synthetically [152]. However, the polypharmacology of synthetic oestrogens, as observed by Perlstein, complicates the identification of specific drug targets and requires careful evaluation to optimise their application in modulating mitochondrial health [153].

In contrast, semi-synthetic drugs such as amitozyn have shown promise in modulating mitochondrial function through mechanisms such as disruption of microtubule polymerisation [154]. This compound has been shown to induce apoptosis in cancer cells, highlighting its potential in targeting mitochondrial pathways for therapeutic purposes. The semi-synthetic modification of natural products can enhance their potency and specificity, bridging the gap between the therapeutic benefits of natural compounds and the precision of synthetic drugs.

The integration of synthetic, semi-synthetic, and plant-derived compounds offers a novel approach to the development of innovative therapeutic strategies. The combination of these categories can enhance treatment efficacy while minimising side effects. Using natural products as templates for synthetic modifications facilitates the creation of drugs that maintain the beneficial properties of their natural counterparts while improving pharmacokinetic and pharmacodynamic profiles [155]. This strategy is exemplified by the synthesis of modified histones and other bioactive compounds, where natural backbones serve as scaffolds for the development of potent derivatives [156].

In addition, advances in biosynthesis and metabolic engineering can greatly enhance the production of bioactive phytochemicals, increasing their availability for therapeutic applications [155]. This is particularly relevant in the fight against drug resistance, where the natural diversity of plant metabolites provides alternative pathways for drug development [157]. Exploring the interactions between synthetic, semi-synthetic, and plant-derived compounds opens up promising avenues for the development of multi-target therapies to effectively modulate mitochondrial health.

The role of mitochondrial health in diseases such as cancer and neurodegenerative disorders highlights the importance of developing drugs that effectively target mitochondrial pathways. For example, selective inhibition of mitochondrial DNA polymerase gamma has been proposed as a strategy to treat certain types of cancer, demonstrating the therapeutic potential of both synthetic and semi-synthetic drugs in targeting mitochondrial function [158].

The modulation of mitochondrial health by synthetic, semi-synthetic, and plant secondary metabolites represents a multifaceted approach to drug development. Each category offers distinct advantages, and their integration promises more effective and safer therapeutic options. Continued research into the mechanisms of action and interactions of these compounds will undoubtedly pave the way for innovative treatments that harness the strengths of both natural and synthetic products.

## 5. Limitations and Risks in the Utilisation of PSMs

The use of plant-derived compounds in medicine has received considerable attention due to their diverse therapeutic properties. However, there is an urgent need to address the potential adverse effects and risks associated with these compounds, particularly their impact on mitochondrial function.

A major concern relates to plant extracts containing saponins, which have been shown to disrupt cellular respiration and mitochondrial membrane potential. For example, components of *Caulophyllum thalictroides* (blue cohosh) have been reported to induce apoptosis by permeabilising mitochondrial membranes, leading to cytotoxic effects in cultured cells [159]. These findings suggest that while saponins may have therapeutic properties, their potential to impair mitochondrial function is a critical safety concern.

Cannabinoids, such as THC and CBD, also affect mitochondrial function. Studies have shown that these compounds can alter the activity of the mitochondrial respiratory chain and increase the production of ROS, thereby compromising cellular integrity and contributing to toxicological effects [145]. Furthermore, CBD has been implicated in impairing mitochondrial metabolism in the brain, raising concerns about its neurotoxic potential [144]. These findings highlight the need for careful characterisation of cannabis-derived extracts, as their therapeutic benefits must be balanced against the risk of mitochondrial dysfunction.

In addition to saponins and cannabinoids, other plant extracts have been associated with mitochondrial toxicity. For example, *Morinda citrifolia* has demonstrated protective effects against drug-induced mitochondrial dysfunction, suggesting a dual potential to both mitigate and contribute to mitochondrial toxicity under different conditions [160]. Similarly, the neuroprotective properties of *Centella asiatica* have been linked to its ability to attenuate oxidative stress and support mitochondrial function, highlighting the complex interplay whereby some phytochemicals may exert protective effects while others pose risks [161].

Levodopa (L-DOPA) is a well-established treatment for PD. However, its therapeutic benefits may be overshadowed by potential neurotoxic effects, particularly those resulting from oxidative stress and mitochondrial dysfunction. Chronic administration of L-DOPA has been associated with increased oxidative stress, which contributes to mitochondrial dysfunction [162,163,164]. Recent studies by Wang et al. showed that L-DOPA stabilised ΔΨm and mitochondrial structure in a rat model of cerebral I/R injury. This duality highlights the need for a nuanced understanding of the role of L-DOPA in mitochondrial interactions, particularly in the context of neurodegeneration [165].

Furthermore, the toxicity of certain phytochemicals may be exacerbated when combined with other phytochemicals. Synergistic interactions between bioactive phytochemicals can enhance therapeutic efficacy but also potentiate mitochondrial damage, increasing the risk of adverse outcomes [159]. This highlights the need for a comprehensive understanding of the interactions between plant-derived compounds and their cumulative effects on mitochondrial health.

The extraction and preparation methods of plant compounds also contribute to the variability in their safety and efficacy. Factors such as extraction techniques, choice of solvent, and the specific plant parts used can influence the concentration and bioactivity of the resulting compounds [166]. This variability complicates standardisation efforts and dosage predictability, making comprehensive risk assessment essential for medicinal applications.

The potential for allergic reactions and other adverse effects associated with herbal medicines warrants careful consideration. While many individuals experience minimal side effects, hypersensitivity or allergic reactions to specific phytochemicals may occur in susceptible individuals [167]. This variability highlights the importance of personalised approaches to herbal medicine use, particularly for individuals with known sensitivities or allergies.

The growing popularity of PMSs also raises concerns about the quality and purity of these products. The largely unregulated market for herbal supplements increases the risk of contamination with heavy metals, pesticides, or other harmful substances [168]. Such contaminants can pose significant health risks, especially when considering the cumulative effects of long-term use.

In addition, the potential for drug interactions is a critical concern. Many plant-derived compounds affect the metabolism of drugs, potentially altering plasma drug concentrations and therapeutic efficacy [169]. For example, the use of *Hypericum perforatum* (St. John’s wort), a widely used herbal remedy, has been associated with significant interactions with drugs such as antidepressants and anticoagulants, potentially leading to serious adverse outcomes.

The therapeutic use of plant-derived compounds requires a comprehensive understanding of their pharmacokinetic and pharmacodynamic properties. The bioavailability of these compounds is influenced by factors such as formulation, route of administration, and individual metabolic variability [170]. Such variability complicates the prediction of therapeutic efficacy and the assessment of potential adverse effects.

Despite the recognised benefits of botanicals, there is an urgent need for rigorous clinical trials to comprehensively assess their safety and efficacy. To date, much of the research in this area has been conducted in vitro or in animal models, which may not fully replicate human physiological responses [171]. In the absence of robust clinical evidence, there is a risk that the safety and efficacy of these compounds may be overestimated, potentially compromising the health of patients.

While plant phytochemicals offer promising therapeutic avenues, particularly in the context of mitochondrial function, their use is associated with a number of risks and potential side effects. The complex biochemistry of plants, coupled with individual variability in response and the possibility of drug interactions, underscores the need for cautious and informed use in medical practice. Future research should prioritise elucidating the mechanisms of action of these compounds, establishing their safety profiles, and developing standardised guidelines for their clinical use.

## 6. Conclusions

This review comprehensively highlights the potential of PSMs, including alkaloids, terpenoids, polyphenols, and compounds derived from whole plant extracts, to improve mitochondrial function and health (see Supplementary Table S1 [172,173,174,175,176,177,178,179,180,181,182,183,184,185,186,187,188,189,190,191,192,193,194,195,196,197,198,199,200,201,202,203,204,205,206,207,208,209,210,211,212,213,214,215,216,217,218,219,220,221,222,223,224,225,226,227,228,229,230,231,232,233,234,235,236,237,238,239,240,241,242,243,244]).

Specific PSMs such as berberine, tannic acid, resveratrol, magnolol, and fisetin have been shown to activate key signalling pathways that promote mitochondrial biogenesis. This activation involves the upregulation of critical regulators, including PGC-1α, Nrf2, TFAM, AMPK, and SIRT1, each of which plays a distinct role in controlling this complex process (see Figure 2). Through the coordinated expression of nuclear and mitochondrial genes, these pathways collectively regulate mitochondrial DNA (mtDNA) replication, transcription, and assembly of the electron transport chain (ETC). For example, resveratrol activates the SIRT1 pathway, which subsequently stimulates PGC-1α expression. As a key regulator of mitochondrial biogenesis, PGC-1α drives the production of new mitochondria, thereby increasing cellular energy capacity.

In addition to biogenesis, PSMs such as panax japonicus, silibinin, carnosic acid, and quercetin have been shown to regulate mitochondrial dynamics by modulating the expression of proteins involved in fusion and fission processes. These metabolites increase the expression of mitofusins (Mfn1 and Mfn2) and OPA1, thereby facilitating mitochondrial fusion (see Figure 2), which is critical for producing healthier and more efficient mitochondria. At the same time, PSMs reduce the activity of proteins involved in mitochondrial fission, such as Drp1, Fis1, and Mff, ensuring the efficient segregation and removal of damaged mitochondria.

Mitophagy, the selective degradation of dysfunctional mitochondria, is another key process modulated by PSMs. These compounds, including DTCA, ETCA, astragaloside IV, silibinin, hesperetin, and berberine, increase the expression of mitophagy-related proteins such as PINK1 and Parkin (see Figure 2), thereby promoting mitochondrial quality control. Conversely, astaxanthin (AST) has been shown to have the opposite effect by reducing mitophagy.

This review also examines the effects of whole plant extracts, demonstrating their multi-pathway effects on mitochondrial function due to their diverse phytochemical profiles. Recent innovations, such as the development of semi-synthetic derivatives, including fluorescently labelled berberine and mito-magnolol, further extend the therapeutic potential of PSMs by improving bioavailability and specificity. As our understanding of these metabolites deepens, their integration into therapeutic strategies offers promising opportunities for novel treatments targeting mitochondrial dysfunction and associated pathologies.

Despite this progress, several challenges remain. Systematic mapping of molecular pathways, particularly the PINK1/Parkin axis, is required to elucidate the precise mechanisms of PSM action. Improved extraction and isolation methods are needed to improve the reproducibility and scalability of studies involving complex plant matrices. In addition, rigorous clinical trials are essential to validate the therapeutic potential of these compounds. Comparative analyses evaluating the efficacy of isolated compounds versus whole plant extracts are also needed to clarify the role of individual phytochemicals and their synergistic interactions within complex matrices.

A unique advantage of PSMs lies in their inherent complexity. These compounds exhibit high compatibility with human physiology, having co-evolved with biological systems over time. This evolutionary compatibility may contribute to improved safety profiles and reduced adverse effects, which often pose challenges for synthetic compounds.

In conclusion, PSMs offer a promising approach to modulating mitochondrial function, with potential applications in the treatment of conditions associated with mitochondrial dysfunction, including neurodegenerative disorders, metabolic syndromes, and cardiovascular diseases. The integration of traditional phytochemical knowledge with modern pharmacological research holds considerable potential for the development of targeted therapies.

## 7. Methodology

A literature search was conducted using PubMed, Scopus, and Google Scholar databases to identify studies relevant to the topic of plant secondary metabolites (PSMs) and their interactions with mitochondria. The initial searches combined various groups of PSMs with the keyword “mitochondria”. To ensure this review included the most recent findings, the search was refined to include papers published within the last five years. Additionally, publications providing data on the solubility of plant compounds in water, ethanol, and DMSO were included. In instances where specific solubility data were unavailable, we referenced general studies that offer established guidelines on the solubility of plant compound types in the aforementioned solvents.

## Supplementary Materials

The following supporting information can be downloaded at https://www.mdpi.com/article/10.3390/ijms26010380/s1.
