# Supplementary material for: Plant Secondary Metabolites as Modulators of Mitochondrial Health: An Overview of Their Anti-Oxidant, Anti-Apoptotic, and Mitophagic Mechanisms
Source: Int J Mol Sci. 2025 Jan 4;26(1):380. doi: 10.3390/ijms26010380 (PMC11720160; doi:10.3390/ijms26010380)
Supplement: Supplementary file 1 [file ijms-26-00380-s001.zip › ijms-3379163-supplementary.pdf]

International Journal of Molecular Sciences

Supplementary Information

Plant Secondary Metabolites as Modulators of Mitochondrial Health: An Overview of Their Antioxidant, Anti-Apoptotic, and Mitophagic Mechanisms

Julia Anchimowicz [1], Piotr Zielonka [1] and Slawomir Jakiela [1,]*

[1] Department of Physics and Biophysics, Institute of Biology, Warsaw University of Life Sciences, 02-787 Warsaw, Poland; julia_anchimowicz@sggw.edu.pl (J.A.); piotr_zielonka@sggw.edu.pl (P.Z.)
* Correspondence: slawomir_jakiela@sggw.edu.pl (S.J.); +48-22-593-8626;

Table S1. Concentrations and solvents of plant secondary metabolites investigated as experimental modulators of mitochondrial health. Abbreviations: AD, Alzheimer's disease; PD, Parkinson's disease; HD, Huntington's disease.

Type of compound	Active substance	Sources	Concentrations and experimental models	Solvent used in experimental models	Mechanism of action	Condition	Solubility
**Alkaloids**	Berberine **(BBR)**	*Phellodendron, Coptis chinensis, Berberis vulgaris, Berberis aristata, Berberis petiolaris, Rhizoma coptidis, Rhizoma cyperus, Rhizoma rhei*	Cell line:<br>5, 10, 20, 40, 60, 80, 100 µM;<br>for 3, 6, 24 h [1]	-	Induction of mitochondrial biogenesis;<br>Activation of mitophagy [1]	-	Water: 2.1 mg/ml [2]<br>Ethanol: 2.75 mg/ml [2]<br>DMF: 0.5 mg/ml [3]<br>DMSO: 25 mg/ml [3]
			Cell line:<br>1 µM; for 24 h;<br>Mice:<br>100 mg/kg/d;<br>orally;<br>for 30 days [4]	PBS [4]	Improvement of mitochondrial functions;<br>Promotion of mitophagy [4]	AD	

			Cell line:<br>2, 4, 8, 16, 32 μM;<br>for 6 h [5]	DMSO [5]	Regulation of mitophagy [5]	PD	
			Rats:<br>150 mg/kg b.w.;<br>intragastrically;<br>for 21 days [6]	Saline [6]	Increase in the activity of mitochondrial complexes [6]	Acetampirid toxicity	
			Rats:<br>200 mg/kg b.w.;<br>orally;<br>for 42 weeks [7]	0.9% saline [7]	Reduction of oxidative stress;<br>Increase in the activity of mitochondrial complexes [7]	Aging	
			Rats:<br>30, 100 mg/kg;<br>orally;<br>for 21 days [8]	Double-distilled water [8]	Reduction of oxidative stress [8]	PD	
	Caffeine **(Cof)**	*Coffea*, Tea, Cocoa	*C. elegans*:<br>10 mM;<br>for 3 days [9]	Nematode growth medium [9]	Regulation of ΔΨm and oxidative stress;<br>Improvement of mitochondrial morphology [9]	Aging	Water: 27.1 mg/ml [10]<br>Ethanol: worse than in water [11]<br>DMSO: better than in water [11]
			Cell line:<br>256, 512 μM;<br>for 1, 24 h [12]	-	Reduction of oxidative stress;<br>Inhibition of mitochondrial apoptosis pathway [12]	UV irradiation	

	Capsaicin **(CS)**	Chili peppers	<u>Cell lines</u>:<br>20, 50, 100 µM;<br>for 1 h [13]	DMSO [13]	Decrease in mitochondrial respiration and ATP production [13]	Lung cancer	Water: poor solubility [14]<br>Ethanol: 61 mg/ml [14]<br>DMSO: 61 mg/ml [14]
			<u>Cells</u>:<br>5, 10, 20, 40, 80 µM;<br>for 12 h [15]	-	Reduction of oxidative stress;<br>Regulation of mitochondrial respiration [15]	LPS-induced sepsis	
	Piperine **(PIP)**	*Piper longum*	<u>Cell line</u>:<br>10, 20, 40 mg/l;<br>for 24 h [16]	Medium [16]	Induction of oxidative stress;<br>Induction of mitochondrial apoptosis pathway [16]	Gastric cancer	Water: 40 mg/l [17]<br>Ethanol: 66.6 mg/ml [17]<br>DMSO: high solubility [17]
			<u>Rats</u>:<br>10 mg/kg;<br>orally;<br>for 15 days [18]	-	Reduction of oxidative stress;<br>Inhibition of mitochondrial apoptosis pathway [18]	Ischemic stroke	
**Phenols**	L-DOPA	*Mucuna pruriens var. Utilize* (leaves, seeds)	<u>Rats</u>:<br>50 mg/kg/d;<br>intraperitoneally;<br>for 7, 14 days [19]	Saline [19]	Stabilisation of ΔΨm and mitochondrial structure [19]	Cerebral ischemia/ reperfusion injury	Water: 10 mg/ml [20]<br>Ethanol: better solubility than in water [20]<br>DMSO: high solubility [20]
**Terpenoids**	Perillaldehyde **(PAE)**	*Perilla frutescens*	<u>*C. elegans*</u>:<br>50, 100 µM [21]	DMSO [21]	Preservation of mitochondrial network [21]	HD	Data is not directly available;<br>Water: limited solubility [22]<br>Ethanol: high solubility [22]<br>DMSO: high solubility [22]

	Asiatic acid (**AA**)	*Centella asiatica*	Mice:<br>0.05% AA = 5 mg AA in 1kg diet;<br>0.5% AA = 50 mg AA in 1 kg diet;<br>1% AA = 1000 mg AA in 1 kg diet [23]	-	Increase in basal mitochondrial respiration [23]	AD	Data is not directly available;<br>Water: poor solubility [24,25]<br>Ethanol: better solubility than in water [24,25]<br>DMSO: better solubility than in water [24,25]
			Mice:<br>100 mg/kg/d;<br>intragastrically;<br>for 7 days [26]	DMSO, saline [26]	Reduction of oxidative stress;<br>Maintenance of mitochondrial morphology [26]	Myocardial ischemia/ reperfusion injury	
			Rats:<br>10, 50 mg/kg;<br>intraperitoneally;<br>for 30 min [27]	1% DMSO [27]	Improvement of mitochondrial functions [27]	Epilepsy	
	α-bisabolol (**BSB**) = levomenol	*Salvia runcinate, Myoporum crassifolium, Matricaria chamomilla, Ereman thusery-thropappus, Siparuna guianensis*	Rats:<br>50 mg/kg b.w./d;<br>intraperitoneally;<br>for 28 days [28]	Sunflower oil [28]	Reduction of oxidative stress;<br>Inhibition of mitochondrial apoptosis pathway [28]	PD	Data is not directly available;<br>Water: poor solubility [29]<br>Ethanol: better solubility than in water [29]<br>DMSO: better solubility than in water [29]

	Astaxanthin (**AST**)	Microalgae	Cell line:<br>20 µM;<br>for 24 h [30]	DMSO [30]	Inhibition of mitochondrial apoptosis pathway;<br>Protection of ΔΨm and ETC proteins [30]	Oxidative stress in neuronal cells	Data is not directly available;<br>Water: poor solubility [31]<br>Ethanol: moderate solubility [31]<br>DMSO: high solubility [31]
		Algae	Cell line:<br>80 µg/l;<br>for 24 h [32]	-	Reduction of mitophagy [32]	Oxidative stress in neuronal cells	
	Forskolin (**FSK**)	*Coleus forskohlii*	Rats:<br>15, 30, 45 mg/kg;<br>orally;<br>for 21 days [33]	2% ethanol in water [33]	Reduction of oxidative stress;<br>Increase in the activity of mitochondrial complexes [33]	PD	Data is not directly available;<br>Water: poor solubility [34]<br>Ethanol: moderate solubility [34]<br>DMSO: high solubility [34]
	Carvacrol (**CARV**)	*Lamiaceae*	Rats:<br>25, 50, 100 mg/kg/d;<br>orally;<br>for 40 days [35]	-	Reduction of oxidative stress;<br>Protection of ΔΨm [35]	Lead toxicity	Water: 0.83 g/l [36]<br>Ethanol: high solubility [37]<br>DMSO: high solubility [38]
			Cell line:<br>100 µM;<br>for 1, 24 h [39]	Medium [39]	Reduction of oxidative stress;<br>Protection of ΔΨm [39]	Oxidative stress in neuronal cells	

	Carnosic acid (**CA**)	*Rosmarinus officinalis, Salvia officinalis*	Mice:<br>30, 60 mg/kg b.w.;<br>orally;<br>for 14 days [40]	1% DMSO [40]	Reduction of oxidative stress [40]	Chlorpyrifos (CPF) toxicity	Data is not directly available;<br>Water: poor solubility [41]<br>Ethanol: better solubility than in water [42]<br>DMSO: better solubility than in water [40,43]
			Cell line:<br>1 μM;<br>for 0.5, 1, 3,18, 24 h [43]	DMSO [43]	Enhancement of mitochondrial fusion;<br>Inhibition of mitochondrial apoptosis pathway [43]	PD	
			Cell line:<br>1 μM;<br>for 18 h [44]	DMSO [44]	Induction of mitochondrial biogenesis [44]	PD	
	Linalool (**LIN**)	Jasmine, basil, rosewood, lavender, thyme	Cells:<br>10, 25, 50 mM;<br>for 4 h [45]	0.05% DMSO [45]	Reduction of oxidative stress;<br>Regulation of ΔΨm [45]	Benzene toxicity	Water: 683.7 mg/L [46]<br>Ethanol: moderate solubility [47]<br>DMSO: high solubility [48]
	Genipin	(poly)phenol-enriched fraction (PEF) of *Corema album* leaf extract	Cells:<br>10 μM;<br>for 24 h [49]	H_2O, acetonitrile with 0.1% formic acid [49]	Modulation of oxidative phosphorylation [49]	PD	Water: less than 1mg/ml [50]<br>Ethanol: moderate solubility [51]<br>DMSO: potential solubility [52]

		Gardenia jasminoides (fruit)	Mice:<br>10 mmol/l = 0.4 µl/eye; intraocularly;<br>Cell line:<br>to 1 µmol/l;<br>for 1-14 days [53]	Penicillin-streptomycin solution (PBS) [53]	Reduction of oxidative stress [53]	Diabetic retinopathy	
**Terpenoids (saponins)**	*P. grandiflorum* crude saponin **(PGS)**	*Platycodon grandiflorum* (roots)	Cell line:<br>5, 10, 20 µg/ml;<br>for 24 h [54]	Ethanol, distilled water [54]	Reduction of oxidative stress;<br>Inhibition of mitochondrial apoptosis pathway [54]	AD	Data is not directly available;<br>Water: poor solubility [55]<br>Ethanol: high solubility [55]<br>DMSO: potential solubility [55]
	P. japonicus saponins **(SPJ)**	*Panax japonicus* (roots)	Rats:<br>10, 30 mg/kg/d;<br>orally;<br>for 4 months;<br>Cell line:<br>25, 50 µg/ml;<br>for 12 + 48 h [56]	Water [56]	Improvement of mitochondrial morphology;<br>Regulation of mitochondrial dynamics;<br>Reduction of oxidative stress [56]	Aging	Data is not directly available;<br>Water: high solubility [57]<br>Ethanol: high solubility [57]<br>DMSO: potential solubility [55]
	Marsdenia Tenacissima saponins **(SMT)**	*Marsdenia Tenacissima*	Cell line:<br>125, 250, 500 µg/ml;<br>for 24 h [58]	PBS [58]	Destruction of mitochondrial morphology;<br>Induction of mitochondrial apoptosis pathway [58]	Liver cancer	Data is not directly available;<br>Water: high solubility [59]<br>Ethanol: high solubility [60]<br>DMSO: potential solubility [55]

	Deoxytrillenoside CA (**DTCA**), Epitrillenoside CA (**ETCA**)	*Trillium tschonoskii*	<u>*C. elegans*</u>:<br>25, 50, 100, 200 μM;<br>for 24, 48, 72 h [61]	-	Reduction of oxidative stress;<br>Induction of mitophagy [62]<br>Promotion of mitochondrial biogenesis [61]	HD	Data is not directly available;<br>Water: poor solubility [62]<br>Ethanol: potential solubility [63]<br>DMSO: high solubility [55]
	Ginsenoside Rb1	*Panax ginseng*	<u>Cells</u>:<br>0.1, 1, 10 μM;<br>for 4 h [64]	Saline [64]	Reduction of oxidative stress;<br>Inhibition of complex I activity [64]	Brain ischemia	Data is not directly available;<br>Water: limited solubility [65]<br>Ethanol: moderate solubility [66]<br>DMSO: high solubility [66]
			<u>Cells</u>:<br>10 μM;<br>for 1 h, for 15 min;<br><u>Mice</u>:<br>50 mg/kg;<br>Intraperitoneally;<br>for 14, 28 days [67]	-	Reduction of oxidative stress;<br>Stabilisation of mPTP and $\Delta\Psi m$;<br>Inhibition of mitochondrial apoptosis pathway [67]	Ischemia/ reperfusion injury	
	Astragaloside IV (**AS-IV**)	*Astragalus membraneaceus*, *Astragalus mongholicus*	<u>Mice</u>:<br>100 mg/kg;<br>intraperitoneally;<br>for 5 weeks [68]	Saline [68]	Reduction of oxidative stress;<br>Promotion of mitophagy [68]	PD	Water: < 1 mg/ml [69]<br>Ethanol: moderate solubility [70]<br>DMSO: high solubility [71]

			Rats:<br>40, 80 mg/kg/d;<br>by gavage;<br>for 28 days [72]	Saline [72]	Improvement of mitochondrial ultrastructure;<br>Promotion of mitophagy;<br>Inhibition of mitochondrial apoptosis pathway [72]	Myasthenia gravis	
			Mice:<br>20 mg/kg/d;<br>for 6 weeks;<br>Cell line:<br>50, 100 μM;<br>for 24 h [73]	-	Reduction of oxidative stress;<br>Improvement of mitochondrial morphology;<br>Enhanced mitochondrial biogenesis [73]	Diabetic nephropathy	
			Rats:<br>30, 90 mg/kg/d;<br>intraperitoneally;<br>for 16-20 months [74]	1% sodium carboxyl methylcellulose [74]	Increase in activities of complexes I, II, III, and IV [74]	Aging	
			Rats:<br>60 mg/kg/d;<br>intragastrically;<br>for 12 weeks [75]	Saline [75]	Improvement of mitochondria morphology;<br>Reduction of oxidative stress;<br>Increase in activities of complexes I, II, III and IV [75]	Diabetic peripheral neuropathy	

	Cycloastragenol (**CAG**)	*Astragalus radix*	Mice:<br>20 mg/kg/d;<br>for 6 weeks [76]	5% DMSO, 2% Tween-20 in PBS [76]	Reduction of oxidative stress;<br>Inhibition of mitochondrial apoptosis pathway [76]	AD	Data is not directly available;<br>Water: poor solubility [77]<br>Ethanol: moderate solubility [78]<br>DMSO: high solubility [71]
			Mice:<br>5, 10, 20 mg/kg;<br>intraperitoneally;<br>for 3 days [79]	DMSO, 2% Tween-20 in PBS [79]	Inhibition of mitochondrial apoptosis pathway [79]	Ischemic stroke	
**Polyphenols (extra-virgin olive oil (EVOO))**	Oleuropein aglycone (**OleA**), Oleuropein (**Ole**), Hydroxytyrosol (**HT**)	Olive leaves and drupes	Cell line:<br>75 μM EVOO;<br>(2/1, 1/1, 1/2) OleA/HT molar ratios;<br>for 24 h [80]	OleA - DMSO;<br>HT – water [80]	Reduction of oxidative stress [80]	AD	Data is not directly available;<br>Water: poor solubility [81]<br>Ethanol: moderate solubility [82]<br>DMSO: likely high solubility [83]
**Polyphenols (flavonoids)**	Quercetin	Common fruits and vegetables (e.g., onions, broccoli, apples, capers, gigs, cranberries)	Rats:<br>50 mg/kg; orally;<br>for 14 days [84]	Saline [84]	Reduction of oxidative stress [84]	PD	Water: 10 mg/l [85]<br>Ethanol: high solubility [86]<br>DMSO: > 100 mg/ml [87]
			Cell line:<br>1, 10, 20, 50, 100 μM;<br>for 4 + 24 h;<br>Rats:<br>30 mg/kg;<br>for 14 days [88]	DMSO [88]	Reduction of oxidative stress;<br>Improvement of mitochondrial structure;<br>Induction of mitophagy [88]	PD	

			Cell line:<br>20, 40, 80 μM;<br>for 24 h [89]	-	Reduction of oxidative stress;<br>Regulation of mitochondrial dynamics [89]	Alcoholic liver disease	
	Naringenin (**NAR**)	Citrus fruits (grapefruit, oranges, bergamot), tomatoes, a few varieties of figs	Cell line:<br>12.5, 20, 25 μM;<br>for 24 h;<br>*Danio rerio*:<br>10, 20, 40 μM;<br>for 72 h [90]	DMSO [90]	Cell line:<br>reduction of oxidative stress;<br>*Danio rerio*:<br>regulation of PD-related genes [90]	PD	Water: 500 mg/l [85]<br>Ethanol: 4.38 μg/ml [91]<br>DMSO: higher solubility than in water [92]
			Cell line:<br>20, 40, 60, 80, 100 μM;<br>for 24 h;<br>Rats:<br>40 mg/kg b.w.;<br>orally;<br>for 21 days [93]	Saline [93]	Reduction of oxidative stress [93]	PD	
			Cell line:<br>100, 200, 400, 800 μM;<br>for 24, 48, 72 h [94]	-	Promotion of mitochondrial apoptosis pathway [94]	Lung cancer	
			Mice:<br>100 mg/kg/d;<br>orally;<br>for 10 months [95]	Carbox-ymethyl-cellulose sodium solution [95]	Regulation of mitochondrial dynamics [95]	Aging	

	Fisetin, quercetin, apigenin, chrysin, naringenin	Fruits, vegetables	<u>*C. elegans*</u>:<br>100 μM;<br>for 15 days [96]	DMSO [96]	Mitochondrial uncoupling [96]	Aging	
	Sterubin, fisetin	Sterubin – *Eriodictyon californicum*;<br>Fisetin – strawberries;	<u>Cell line</u>:<br>sterubin – 2.5 μM;<br>fisetin – 5 μM;<br>for 16 h (overnight) [97]	0.2% ethanol	Reduction of oxidative stress;<br>Regulation of mitochondrial dynamics;<br>Enhancement in mitochondrial bioenergetics [97]	Ferroptosis	
	Gossypitrin **(Gos)**	*Talipariti elatum*	<u>Cell line, cells</u>:<br>0.01, 1, 10, 20, 25, 30, 40, 50, 60, 80, 100 μM;<br>for 2, 24 h [98]	DMSO [98]	Reduction of oxidative stress;<br>Preservation of mitochondrial morphology [98]	Iron toxicity	Data is not directly available;<br>Water: moderate solubility [99]<br>Ethanol: moderate solubility [99]<br>DMSO: moderate solubility [98]

	Diosmin (**DSM**)	Pericarp of citrus fruits (*Rutaceae*)	Rats:<br>50, 100 mg/kg;<br>intraperitoneally;<br>for 21 days [100]	0.5% DMSO, normal saline [100]	Reduction of oxidative stress;<br>Enhancement of complex I/II activity [100]	Neurodegeneration	Data is not directly available;<br>Water: poor solubility [101]<br>Ethanol: moderate solubility [101]<br>DMSO: better solubility than in ethanol [102]
	Silibinin (**SIL**)	*Silybum marianumis* (fruit), *Silybum marianum* (seeds)	Mice:<br>70, 140, 280 mg/kg;<br>orally;<br>for 7 days [103]	0.5% sodium carboxymethyl cellulose [103]	Reduction of oxidative stress;<br>Promotion of mitophagy [103]	PD	Water: 0.4 mg/100 ml [104]<br>Ethanol: poor solubility [105]<br>DMSO: high solubility [105]
			Mice:<br>70, 140, 280 mg/kg/d;<br>intragastrically;<br>for 24 days [106]	0.5% sodium carboxymethyl cellulose [106]	Reduction of oxidative stress;<br>Inhibition of mitochondrial apoptosis pathway;<br>Regulation of mitochondrial dynamics and morphology [106]	PD	
			Cell line:<br>1, 5, 10 μM;<br>for 12 h [107]	Medium [107]	Reduction of oxidative stress;<br>Regulation of mitochondrial dynamics and morphology [107]	Oxidative stress in neuronal cells	

			Cell line:<br>50, 100 μM;<br>for 24 h [108]	DMSO<br>[108]	Inhibition of mitochondrial rupturing [108]	AD	
			Cell line:<br>50, 100 μM;<br>for 24 h [109]	0.1%<br>DMSO<br>[109]	Induction of mitochondrial apoptosis pathway [109]	Oral cancer	
	Luteolin (**LUT**)	Vegetables,<br>fruits,<br>herbs,<br>*Ajuga decumbens thumb*	Cell line:<br>1, 2.5, 5 μM;<br>for 6, 24 h [110]	DMSO<br>[110]	Enhancement of mitochondrial complexes I and II activities;<br>Increase in the number of contacts between mitochondria and ER [110]	-	Water: 0.4 mg/l [85]<br>Ethanol: moderate solubility [111]<br>DMSO: high solubility [85]
			Cells:<br>1, 10, 50 μM;<br>for 24 h [112]	DMSO<br>[112]	Decrease in apoptosis;<br>Promotion of mitochondrial autophagy [112]	Cardiotoxicity	
			Cell line:<br>10, 20, 30, 40, 50, 60, 70, 80 μM;<br>for 24, 48 h [113]	DMSO, complete medium<br>[113]	Promotion of mitochondrial apoptosis pathway;<br>Disruption of mitochondrial functions [113]	Gastric cancer	
			Cells:<br>0.5 μg/ml;<br>for 12 + 24 h [114]	Medium<br>[114]	Regulation of ATP production and mitochondrial dynamics [114]	Ammonia poisoning	

**Polyphenols (flavonoid-rich extract)**	Mandarin juice extract (MJe)	*Citrus reticulata*	Cell line:<br>0.1, 0.5 mg/ml;<br>for 1, 24 h [115]	DMSO [115]	Reduction of oxidative stress;<br>Downregulation of PD-related genes;<br>Inhibition of mitochondrial apoptosis pathway [115]	PD	Data is not directly available;<br>Water: high solubility [116]<br>Ethanol: high solubility [116]<br>DMSO: moderate solubility [115]
**Polyphenols (flavonoids)**	Hesperidin (**Hsd**)	Citrus fruits (e.g., oranges, grapefruit, lemons, tangerines)	Mice:<br>50 mg/kg/d;<br>orally;<br>for 28 days [117]	Saline [117]	Increase in mitochondrial enzymes activity;<br>Inhibition of mitochondrial apoptosis pathway [117]	PD	Water: 4.93 μg/ml [118]<br>Ethanol: high solubility [119]<br>DMSO: high solubility [119]
	Hesperetin (**Hst**)		Cell lines:<br>25, 50,100, 200, 300, 400, 600, 800 μM;<br>for 24, 48 h [120]	DMSO [120]	Enhancement of oxidative stress;<br>Induction of mitochondrial apoptosis pathway [120]	Bladder cancer	Water: 4.5 μmol/l [121]<br>Ethanol: 87 mmol/l [121]<br>DMSO: 5 mg/ml [122]
			Cell line:<br>20, 40 μM;<br>for 4, 24 h [123]	0.1% DMSO [123]	Improvement of mitochondrial functions;<br>Promotion of mitophagy [123]	Nonalcoholic fatty liver disease	

	Epigallocatechin gallate (**EGCG**), gallocatechin gallate (**GCG**), gallocatechin (**GC**), epicatechin gallate (**ECG**), epicatechin (**EC**)	Green tea (young leaves) extract of Camellia (*Camellia sinensis*)	Cell line:<br>Catechins:<br>3.15, 6.25, 12.5, 25, 50, 100, 200 μM;<br>GCG:<br>50, 100 μM [124]	DMSO [124]	Reduction of oxidative stress [124]	Oxidative stress in neuronal cells	Data is not directly available;<br>Water: partial solubility [125]<br>Ethanol: high solubility [125]<br>DMSO: high solubility [124]
	Icariin (**ICA**)	*Epimedium brevicornu, E. wushanense, E. sagittatum, E. pubescens*	Cell line:<br>5 μmol/l for 24 h +<br>5 μmol/l for 48 h [126]	DMSO [126]	Inhibition of mitochondria-related autophagy [126]	Neuronal injury	Water: 15 μg/ml [127]<br>Ethanol: 188 mg/l [128]<br>DMSO: high solubility [126,129]
			Cells:<br>0.1, 1, 10 μM;<br>for 24 h [130]	Medium [130]	Reduction of oxidative stress;<br>Inhibition of mitochondrial apoptosis pathway [130]	Intervertebral disc degeneration	
			Cell line:<br>10, 20 μM;<br>for 24 h [129];	DMSO, medium [129]	Promotion of mitochondrial apoptosis pathway;<br>Induction of oxidative stress [129]	Breast cancer	

**Polyphenols (phenylpropanoid + flavonoid)**	β-Asarone + ICA	β-Asarone - *Acorus tatarinowii*; ICA - *Epimedium*	Cell line:<br>β-asarone: 36 μM;<br>ICA: 6 μM;<br>for 12 h;<br>Mice:<br>β-asarone: 15 mg/kg/d;<br>ICA: 20 mg/kg/d;<br>for 4 weeks [131]	-	Induction of mitophagy [131];<br>β-Asarone:<br>Ability to cross the blood-brain barrier [132]	AD	β-Asarone :<br>Data is not directly available;<br>Water: poor solubility [133]<br>Ethanol: better solubility than in water [133]<br>DMSO: better solubility than in water [134]
**Polyphenols (nonflavonoid polyphenols)**	Magnolol (**MGN**)	*Magnolia officinalis*, *Magnolia grandiflora*	*C. elegans*:<br>2.5, 5, 10, 25, 50 μM;<br>for different time periods [135]	DMSO, *E.coli* OP50 [135]	Reduction of oxidative stress;<br>Preservation of mitochondrial morphology [135]	Aging	Water: 12.5 μg/ml [136]<br>Ethanol: better solubility than in water [137]<br>DMSO: better solubility than in water [135]
			Cells:<br>4 μM;<br>for 12 h [138]	-	Reduction of oxidative stress;<br>Elevation of mitochondrial mass and ΔΨm [138]	Osteoarthritis	
	Mito-magnolol (**Mito-MGN**)	Semisyntetic	Cell line:<br>0.01, 0.03, 0.1, 0.2, 0.3, 0.4, 0.5, 0.75, 1, 3, 10 μM;<br>for 24, 48 h [139]	20% ethanol in PBS [139]	Inhibition of complex I activity;<br>Induction of mitophagy;<br>Decrease of ΔΨm [139];	Melanoma	Data is not directly available;<br>Water : poor solubility [136]<br>Ethanol: moderate solubility [140]<br>DMSO: moderate solubility [140]

	CT1-3 (MGN-SFN hybrid)	Syntetic	Cell line: 7.5, 10 µM; for 8, 24 h [141]	-	Induction of mitochondrial apoptosis pathway [141]	Cancer	Data is not directly available; Nanocarrier systems and optimizing solvent compositions can significantly enhance solubility [142]
	Tannic acid (**TA**)	Many fruits and vegetables (e.g., grapes), black tea, nuts, sorghums	Rats: 50 mg/kg b.w.; orally; for 28 days [143]	Water [143]	Reduction of oxidative stress; Inhibition of mitochondrial apoptosis pathway [143]	PD	Water: 300 g/l [144]
			Rats: 50 mg/kg b.w.; intraperitoneally; 30 min before and 6 and 18 h after post-TBI [145]	0.9% sterile normal saline [145]	Reduction of oxidative stress; Inhibition of apoptosis pathway; Enhancement of mitochondrial biogenesis [145]	Traumatic brain injury	
			Cell line: 25, 50, 100, 200 µM; for 24 h [146]	Medium [146]	Induction of mitochondrial apoptosis pathway [146]	Bladder cancer	
	Resveratrol (**RSV**)	Grapes, berries, peanuts, herbs	Rats: 10, 30, 50 mg/kg; for 24 h; Cell line: 1, 10, 25, 50, 75, 100 M; for 1 h [147]	DMSO, 0.9% saline [147]	Reduction of oxidative stress; Improvement of mitochondrial morphology and functions [147]	Sepsis-induced cardiomyopathy	Water: 0.05 mg/ml [148] Ethanol: 50 mg/ml [148] DMSO: 16 mg/ml [148]

			Cells:<br>5, 20 μM;<br>for 24 h [149]	DMSO [149]	Reduction of oxidative stress;<br>Elevation of mitochondrial content;<br>Promotion of mitophagy [149]	Myocardial ischemia/ reperfusion injury	
			Carassius gibelio:<br>500 mg/kg;<br>3 times/day;<br>for 8 weeks [150]	-	Reduction of oxidative stress;<br>Regulation of mitochondrial dynamics [150]	Lipopolysaccharide (LPS)-induced liver injury	
			Cell line:<br>20 μM; for 2 h;<br>Rats:<br>200 mg/kg b.w./d;<br>by gavage;<br>for 50 days [151]	Water [151]	Enhancement of mitochondrial biogenesis;<br>Improvement of mitochondrial morphology [151]	Spontaneous intracerebral hemorrhage	
			Drosophila melanogaster:<br>15, 30, 60 mg/kg diet;<br>for 10 days [152]	2% ethanol [152]	Reduction of oxidative stress;<br>Elevation of mitochondrial content [152]	PD	
			Cell line:<br>6, 12 μg/ml;<br>for 48 h [153]	DMSO [153]	Reduction of oxidative stress;<br>Activation of mitochondrial apoptosis pathway [153]	Colorectal cancer	

	Pterostilbene (**PTE**)	Blueberry, grapes	Rats:<br>25 mg/kg/d;<br>orally;<br>for 30 days [154]	-	Reduction of oxidative stress [154]	Cerebral ischemia/ reperfusion injury	Water: 21 mg/l [155]<br>Ethanol: 50 g/l [155]<br>DMSO: 16 g/l [155]
			Mice:<br>10 mg/kg;<br>for 72h;<br>Cell line:<br>5 μM;<br>for 4 + 24 h [156]	DMSO, saline [156]	Mice:<br>Inhibition of mitochondrial apoptosis pathway;<br>Cell line:<br>Regulation of mitochondrial dynamics and morphology [156]	Secondary brain injury after intracerebral hemorrhage	
			Cell line:<br>20, 40, 80,100 μM;<br>for 24, 48, 72 h [157]	DMSO [157]	Induction of oxidative stress;<br>Promotion of mitochondrial apoptosis pathway [157]	Glioma	
	Mangiferin (**MGF**)	*Gentianaceae, Mangifera indica, Anemarrhena asphodeloides, Iris unguicularis, Arrabidaea patellifera,* [illegible]	Cell line:<br>10, 25, 50 μM;<br>for 4 h [158]	DMSO [158]	Inhibition of mitochondrial apoptosis pathway;<br>Regulation of mitochondrial dynamics [158];	Fluoride toxicity	Water: 0.111 mg/ml [159]<br>Ethanol: poor solubility [160]<br>DMSO: high solubility [161]
			Cell line:<br>20 μM;<br>for 1 + 24 h [162]	DMSO [162]	Inhibition of mitochondrial apoptosis pathway;<br>Reduction of oxidative stress [162]	Oxidative stress	

			Mice:<br>10, 20, 40 mg/kg b.w.;<br>intraperitoneally;<br>twice/day for 7 days + once/day for 5 days [163]	2% Tween-80<br>in saline<br>[163]	Regulation of mitochondrial morphology, dynamics and functions;<br>Induction of mitophagy [163]	PD	
			Cells:<br>100, 500 μM/ml;<br>for 24, 48 h [164]	PBS [164]	Improvement of mitochondrial morphology and functions;<br>Inhibition of mitochondrial apoptosis pathway [164]	Intervertebral disc degeneration	
**Polyphenols (ellagitannins)**	Urolithin A (UA)	Pomegranate, nuts, strawberries, berries	Cell line:<br>1, 10 μM;<br>for 24 h [165]	DMSO [165]	Enhancement of OXPHOS and mitochondrial biogenesis [165]	AD	Data is not directly available;<br>Water: poor solubility [166]<br>Ethanol: high solubility [166]<br>DMSO: high solubility [166]
			Cell line:<br>2, 10, 20, 30 μM;<br>for 2, 24 h;<br>Tissues:<br>30 μM;<br>for 2 h [167]	-	Induction of mitophagy;<br>Restoration of ΔΨm [167]	Aging	
			Cells:<br>6.25, 12 μM;<br>for 24 h [168]	DMSO [168]	Enhancement of respiration;<br>Induction of mitophagy [168]	Osteoarthritis	

			Humans:<br>500, 1000 mg/d;<br>orally;<br>for 120 days [169]	-	Induction of mitophagy;<br>Stimulation of ETC enzymes activity [169]	Aging	
	UA + EGCG	UA: pomegranates;<br>EGCG: green tea	Mice:<br>UA: 2.5 mg/kg b.w.;<br>EGCG: 25 mg/kg b.w.;<br>intraperitoneally;<br>3 times/week for 4 months [170]	UA – DMSO<br>EGCG – H_2O [170]	Reduction of oxidative stress;<br>Improvement of mitochondrial dynamics and functions [170]	AD	Data is not directly available
**Glucosinolates**	Sulforaphane (**SFN**)	Cruciferous plants<br>(e.g., broccoli, cauliflower, cabbage, kale)	Cell line:<br>0.5, 1, 2, 5 μM;<br>for 6 – 24 h [171]	DMSO [171]	Reduction of oxidative stress;<br>Inhibition of mitochondrial apoptosis pathway;<br>Increase in mitochondrial enzymes activity [171]	CPF and LPS cytotoxicity	Water: 2.55 mg/ml [172]<br>Ethanol: 20 mg/ml [172]<br>DMSO: high solubility [171]
			Rats:<br>5 mg/kg/dose;<br>intraperitoneally;<br>2 doses;<br>24, 48 h [173]	DMSO, PBS [173]	Reduction of oxidative stress [173]	Epilepsy	
			Cells:<br>5 μM;<br>for 72 h [174]	0.01% DMSO [174]	Enhancement of coupling efficiency [174]	Fragile X-Associated Tremor/Ataxia Syndrome	

**Leaves extract**	Ethanolic extract of *Mentha piperita* **(EthMP)**	*Mentha piperita*	Mice:<br>200 mg/kg/d;<br>orally;<br>for 21 days [175]	2.5% DMSO [175]	Reduction of oxidative stress [175]	PD	Data is not directly available;<br>Water: limited solubility [176]<br>Ethanol: high solubility [176]<br>DMSO: high solubility [175]
**Phytocannabinoids**	Cannabinol **(CBN)**	*Cannabis sativa*	Cell line:<br>5, 10 μM;<br>for 1 h + 16 h [177]	0.2% ethanol [177]	Reduction of oxidative stress;<br>Promotion of mitochondrial biogenesis;<br>Regulation of mitochondrial dynamics [177]	Ferroptosis	Data is not directly available;<br>Water: poor solubility [178]<br>Ethanol: high solubility [178]<br>DMSO: high solubility [178]

References

1. Um, J.-H.; Lee, K.-M.; Kim, Y.-Y.; Lee, D.-Y.; Kim, E.; Kim, D.-H.; Yun, J. Berberine Induces Mitophagy through Adenosine Monophosphate-Activated Protein Kinase and Ameliorates Mitochondrial Dysfunction in PINK1 Knockout Mouse Embryonic Fibroblasts. *Int. J. Mol. Sci.* **2023**, *25*, 219, doi:10.3390/ijms25010219.
2. Sut, S.; Faggian, M.; Baldan, V.; Poloniato, G.; Castagliuolo, I.; Grabnar, I.; Perissutti, B.; Brun, P.; Maggi, F.; Voinovich, D.; et al. Natural Deep Eutectic Solvents (NADES) to Enhance Berberine Absorption: An In Vivo Pharmacokinetic Study. *Molecules* **2017**, *22*, 1921, doi:10.3390/molecules22111921.
3. Berberine (Chloride) (CAS 633-65-8) Available online: https://www.caymanchem.com/product/10006427 (accessed on 19 November 2024).
4. Wang, C.; Zou, Q.; Pu, Y.; Cai, Z.; Tang, Y. Berberine Rescues D-Ribose-Induced Alzheimer's Pathology via Promoting Mitophagy. *Int. J. Mol. Sci.* **2023**, *24*, 5896, doi:10.3390/ijms24065896.
5. Wang, L.; Sheng, W.; Tan, Z.; Ren, Q.; Wang, R.; Stoika, R.; Liu, X.; Liu, K.; Shang, X.; Jin, M. Treatment of Parkinson's Disease in Zebrafish Model with a Berberine Derivative Capable of Crossing Blood Brain Barrier, Targeting Mitochondria, and Convenient for Bioimaging Experiments. *Comp. Biochem. Physiol. Part C Toxicol. Pharmacol.* **2021**, *249*, 109151, doi:10.1016/j.cbpc.2021.109151.
6. Phogat, A.; Singh, J.; Malik, V.; Kumar, V. Neuroprotective Potential of Berberine against Acetamiprid Induced Toxicity in Rats: Implication of Oxidative Stress, Mitochondrial Alterations, and Structural Changes in Brain Regions. *J. Biochem. Mol. Toxicol.* **2023**, *37*, e23434, doi:10.1002/jbt.23434.

7. Yadawa, A.K.; Srivastava, P.; Singh, A.; Kumar, R.; Arya, J.K.; Rizvi, S.I. Berberine Attenuates Brain Aging via Stabilizing Redox Homeostasis and Inflammation in an Accelerated Senescence Model of Wistar Rats. *Metab. Brain Dis.* **2024**, *39*, 649–659, doi:10.1007/s11011-024-01350-7.
8. Tseng, H.-C.; Wang, M.-H.; Fang, C.-H.; Lin, Y.-W.; Soung, H.-S. Neuroprotective Potentials of Berberine in Rotenone-Induced Parkinson's Disease-like Motor Symptoms in Rats. *Brain Sci.* **2024**, *14*, 596, doi:10.3390/brainsci14060596.
9. Min, H.; Youn, E.; Shim, Y.-H. Long-Term Caffeine Intake Exerts Protective Effects on Intestinal Aging by Regulating Vitellogenesis and Mitochondrial Function in an Aged Caenorhabditis Elegans Model. *Nutrients* **2021**, *13*, 2517, doi:10.3390/nu13082517.
10. Machmudah, S.; Kitada, K.; Sasaki, M.; Goto, M.; Munemasa, J.; Yamagata, M. Simultaneous Extraction and Separation Process for Coffee Beans with Supercritical CO2 and Water. *Ind. Eng. Chem. Res.* **2011**, *50*, 2227–2235, doi:10.1021/ie101252w.
11. Molajafari, F.; Li, T.; Abbasichaleshtori, M.; D, M.H.Z.; Cozzolino, A.F.; Fandrick, D.R.; Howe, J.D. Computational Screening for Prediction of Co-Crystals: Method Comparison and Experimental Validation. *CrystEngComm* **2024**, *26*, 1620–1636, doi:10.1039/D3CE01252B.
12. Xu, H.; Gan, C.; Gao, Z.; Huang, Y.; Wu, S.; Zhang, D.; Wang, X.; Sheng, J. Caffeine Targets SIRT3 to Enhance SOD2 Activity in Mitochondria. *Front. Cell Dev. Biol.* **2020**, *8*, 822, doi:10.3389/fcell.2020.00822.
13. Han, T.-H.; Park, M.K.; Nakamura, H.; Ban, H.S. Capsaicin Inhibits HIF-1α Accumulation through Suppression of Mitochondrial Respiration in Lung Cancer Cells. *Biomed. Pharmacother.* **2022**, *146*, 112500, doi:10.1016/j.biopha.2021.112500.
14. Capsaicin Available online: https://www.selleckchem.com/datasheet/capsaicin-S199002-DataSheet.html (accessed on 15 November 2024).
15. Qiao, Y.; Wang, L.; Hu, T.; Yin, D.; He, H.; He, M. Capsaicin Protects Cardiomyocytes against Lipopolysaccharide-Induced Damage via 14-3-3γ-Mediated Autophagy Augmentation. *Front. Pharmacol.* **2021**, *12*, 659015, doi:10.3389/fphar.2021.659015.
16. Guo, L.; Yang, Y.; Sheng, Y.; Wang, J.; Ruan, S.; Han, C. Mechanism of Piperine in Affecting Apoptosis and Proliferation of Gastric Cancer Cells via ROS-mitochondria-associated Signalling Pathway. *J. Cell. Mol. Med.* **2021**, *25*, 9513, doi:10.1111/jcmm.16891.
17. Milenković, A.N.; Stanojević, L.P. Black Pepper: Chemical Composition and Biological Activities. *Adv. Technol.* **2021**, *10*, 40–50, doi:10.5937/savteh2102040M.
18. Kaushik, P.; Ali, M.; Salman, M.; Tabassum, H.; Parvez, S. Harnessing the Mitochondrial Integrity for Neuroprotection: Therapeutic Role of Piperine against Experimental Ischemic Stroke. *Neurochem. Int.* **2021**, *149*, 105138, doi:10.1016/j.neuint.2021.105138.
19. Wang, W.; Zhao, J.; Li, Z.; Kang, X.; Li, T.; Isaev, N.K.; Smirnova, E.A.; Shen, H.; Liu, L.; Yu, Y. L-DOPA Ameliorates Hippocampus-Based Mitochondria Respiratory Dysfunction Caused by GCI/R Injury. *Biomed. Pharmacother.* **2024**, *175*, 116664, doi:10.1016/j.biopha.2024.116664.
20. Liu, X.; Han, X.; Peng, Y.; Tan, C.; Wang, J.; Xue, H.; Xu, P.; Tao, F. Rapid Production of L-DOPA by Vibrio Natriegens, an Emerging next-Generation Whole-Cell Catalysis Chassis. *Microb. Biotechnol.* **2022**, *15*, 1610–1621, doi:10.1111/1751-7915.14001.
21. Fang, M.; Liu, Y.; Gao, X.; Yu, J.; Tu, X.; Mo, X.; Zhu, H.; Zou, Y.; Huang, C.; Fan, S. Perillaldehyde Alleviates polyQ-Induced Neurodegeneration through the Induction of Autophagy and Mitochondrial UPR in Caenorhabditis Elegans. *BioFactors Oxf. Engl.* **2024**, doi:10.1002/biof.2089.

22. Ning, J.; Sha, L.; Zuo, Q.; Wei, R.; Sun, C.; Wei, J.; Wang, M. Room-Temperature Stable Perillaldehyde—Natural Cyclodextrin Inclusion Complexes: Preparation, Characterization, Thermal Stability, Water Solubility, Antioxidant Activity and Slow–Release Performance. *J. Mol. Struct.* **2024**, *1312*, 138483, doi:10.1016/j.molstruc.2024.138483.
23. Varada, S.; Chamberlin, S.R.; Bui, L.; Brandes, M.S.; Gladen-Kolarsky, N.; Harris, C.J.; Hack, W.; Brumbach, B.H.; Quinn, J.F.; Gray, N.E. Asiatic Acid Improves Mitochondrial Function, Activates Antioxidant Response in the Mouse Brain and Improves Cognitive Function in Beta-Amyloid Overexpressing Mice 2024, 2024.02.21.581270.
24. Lu, C.-W.; Lin, T.-Y.; Pan, T.-L.; Wang, P.-W.; Chiu, K.-M.; Lee, M.-Y.; Wang, S.-J. Asiatic Acid Prevents Cognitive Deficits by Inhibiting Calpain Activation and Preserving Synaptic and Mitochondrial Function in Rats with Kainic Acid-Induced Seizure. *Biomedicines* **2021**, *9*, 284, doi:10.3390/biomedicines9030284.
25. Soumyanath, A.; Zhong, Y.-P.; Henson, E.; Wadsworth, T.; Bishop, J.; Gold, B.G.; Quinn, J.F. Centella Asiatica Extract Improves Behavioral Deficits in a Mouse Model of Alzheimer's Disease: Investigation of a Possible Mechanism of Action. *Int. J. Alzheimer's Dis.* **2012**, *2012*, 381974, doi:10.1155/2012/381974.
26. Yi, C.; Song, M.; Sun, L.; Si, L.; Yu, D.; Li, B.; Lu, P.; Wang, W.; Wang, X. Asiatic Acid Alleviates Myocardial Ischemia-Reperfusion Injury by Inhibiting the ROS-Mediated Mitochondria-Dependent Apoptosis Pathway. *Oxid. Med. Cell. Longev.* **2022**, *2022*, 3267450, doi:10.1155/2022/3267450.
27. Lu, C.-W.; Lin, T.-Y.; Pan, T.-L.; Wang, P.-W.; Chiu, K.-M.; Lee, M.-Y.; Wang, S.-J. Asiatic Acid Prevents Cognitive Deficits by Inhibiting Calpain Activation and Preserving Synaptic and Mitochondrial Function in Rats with Kainic Acid-Induced Seizure. *Biomedicines* **2021**, *9*, 284, doi:10.3390/biomedicines9030284.
28. Javed, H.; Meeran, M.F.N.; Azimullah, S.; Bader Eddin, L.; Dwivedi, V.D.; Jha, N.K.; Ojha, S. α-Bisabolol, a Dietary Bioactive Phytochemical Attenuates Dopaminergic Neurodegeneration through Modulation of Oxidative Stress, Neuroinflammation and Apoptosis in Rotenone-Induced Rat Model of Parkinson's Disease. *Biomolecules* **2020**, *10*, 1421, doi:10.3390/biom10101421.
29. Tai, Y.; Wang, H.; Yao, P.; Sun, J.; Guo, C.; Jin, Y.; Yang, L.; Chen, Y.; Shi, F.; Yu, L.; et al. Biosynthesis of α-Bisabolol by Farnesyl Diphosphate Synthase and α-Bisabolol Synthase and Their Related Transcription Factors in Matricaria Recutita L. *Int. J. Mol. Sci.* **2023**, *24*, 1730, doi:10.3390/ijms24021730.
30. Brasil, F.B.; Bertolini Gobbo, R.C.; Souza De Almeida, F.J.; Luckachaki, M.D.; Dall'Oglio, E.L.; De Oliveira, M.R. The Signaling Pathway PI3K/Akt/Nrf2/HO-1 Plays a Role in the Mitochondrial Protection Promoted by Astaxanthin in the SH-SY5Y Cells Exposed to Hydrogen Peroxide. *Neurochem. Int.* **2021**, *146*, 105024, doi:10.1016/j.neuint.2021.105024.
31. Liu, Y.; Yang, L.; Guo, Y.; Zhang, T.; Qiao, X.; Wang, J.; Xu, J.; Xue, C. Hydrophilic Astaxanthin: PEGylated Astaxanthin Fights Diabetes by Enhancing the Solubility and Oral Absorbability. *J. Agric. Food Chem.* **2020**, *68*, 3649–3655, doi:10.1021/acs.jafc.0c00784.
32. Yan, T.; Ding, F.; Zhang, Y.; Wang, Y.; Wang, Y.; Zhang, Y.; Zhu, F.; Zhang, G.; Zheng, X.; Jia, G.; et al. Astaxanthin Inhibits H2O2-Induced Excessive Mitophagy and Apoptosis in SH-SY5Y Cells by Regulation of Akt/mTOR Activation. *Mar. Drugs* **2024**, *22*, doi:10.3390/md22020057.

33. Alharbi, M.; Alshammari, A.; Kaur, G.; Kalra, S.; Mehan, S.; Suri, M.; Chhabra, S.; Kumar, N.; Alanazi, W.A.; Alshanwani, A.R.; et al. Effect of Natural Adenylcyclase/cAMP/CREB Signalling Activator Forskolin against Intra-Striatal 6-OHDA-Lesioned Parkinson's Rats: Preventing Mitochondrial, Motor and Histopathological Defects. *Molecules* **2022**, *27*, 7951, doi:10.3390/molecules27227951.
34. Singh, P.; Suryanarayana1, M.A. Effect of Solvents and Extraction Methods on Forskolin Content from Coleus Forskholii Roots. *Indian J. Pharm. Sci.* **2020**, *81*, 1136–1140, doi:10.36468/pharmaceutical-sciences.614.
35. Zare Mehrjerdi, F.; Niknazar, S.; Yadegari, M.; Akbari, F.A.; Pirmoradi, Z.; Khaksari, M. Carvacrol Reduces Hippocampal Cell Death and Improves Learning and Memory Deficits Following Lead-Induced Neurotoxicity via Antioxidant Activity. *Naunyn. Schmiedebergs Arch. Pharmacol.* **2020**, *393*, 1229–1237, doi:10.1007/s00210-020-01866-6.
36. Chang, Y.; McLandsborough, L.; McClements, D.J. Physicochemical Properties and Antimicrobial Efficacy of Carvacrol Nanoemulsions Formed by Spontaneous Emulsification. *J. Agric. Food Chem.* **2013**, *61*, 8906–8913, doi:10.1021/jf402147p.
37. Cerisuelo, J.P.; Gavara, R.; Hernández-Muñoz, P. Antimicrobial-Releasing Films and Coatings for Food Packaging Based on Carvacrol and Ethylene Copolymers. *Polym. Int.* **2015**, *64*, 1747–1753, doi:10.1002/pi.4975.
38. Zhou, S.; Han, C.; Zhang, C.; Kuchkarova, N.; Wei, C.; Zhang, C.; Shao, H. Allelopathic, Phytotoxic, and Insecticidal Effects of Thymus Proximus Serg. Essential Oil and Its Major Constituents. *Front. Plant Sci.* **2021**, *12*, doi:10.3389/fpls.2021.689875.
39. Nazıroğlu, M. A Novel Antagonist of TRPM2 and TRPV4 Channels: Carvacrol. *Metab. Brain Dis.* **2022**, *37*, 711–728, doi:10.1007/s11011-021-00887-1.
40. AlKahtane, A.A.; Ghanem, E.; Bungau, S.G.; Alarifi, S.; Ali, D.; AlBasher, G.; Alkahtani, S.; Aleya, L.; Abdel-Daim, M.M. Carnosic Acid Alleviates Chlorpyrifos-Induced Oxidative Stress and Inflammation in Mice Cerebral and Ocular Tissues. *Environ. Sci. Pollut. Res.* **2020**, *27*, 11663–11670, doi:10.1007/s11356-020-07736-1.
41. Vaka, S.R.K.; Shivakumar, H.N.; Repka, M.A.; Murthy, S.N. Formulation and Evaluation of Carnosic Acid Nanoparticulate System for Upregulation of Neurotrophins in the Brain upon Intranasal Administration. *J. Drug Target.* **2013**, *21*, 44–53, doi:10.3109/1061186X.2012.725405.
42. Cháfer, A.; Fornari, T.; Berna, A.; Ibañez, E.; Reglero, G. Solubility of Solid Carnosic Acid in Supercritical CO2 with Ethanol as a Co-Solvent. *J. Supercrit. Fluids* **2005**, *34*, 323–329, doi:10.1016/j.supflu.2004.10.009.
43. Lin, C.-Y.; Chen, W.-J.; Fu, R.-H.; Tsai, C.-W. Upregulation of OPA1 by Carnosic Acid Is Mediated through Induction of IKKγ Ubiquitination by Parkin and Protects against Neurotoxicity. *Food Chem. Toxicol.* **2020**, *136*, 110942, doi:10.1016/j.fct.2019.110942.
44. Lin, C.-Y.; Huang, Y.-N.; Fu, R.-H.; Liao, Y.-H.; Kuo, T.-Y.; Tsai, C.-W. Promotion of Mitochondrial Biogenesis via the Regulation of PARIS and PGC-1α by Parkin as a Mechanism of Neuroprotection by Carnosic Acid. *Phytomedicine* **2021**, *80*, 153369, doi:10.1016/j.phymed.2020.153369.
45. Salimi, A.; Khodaparast, F.; Bohlooli, S.; Hashemidanesh, N.; Baghal, E.; Rezagholizadeh, L. Linalool Reverses Benzene-Induced Cytotoxicity, Oxidative Stress and Lysosomal/Mitochondrial Damages in Human Lymphocytes. *Drug Chem. Toxicol.* **2022**, *45*, 2454–2462, doi:10.1080/01480545.2021.1957563.

46. Api, A.M.; Belsito, D.; Bhatia, S.; Bruze, M.; Calow, P.; Dagli, M.L.; Dekant, W.; Fryer, A.D.; Kromidas, L.; La Cava, S.; et al. RIFM Fragrance Ingredient Safety Assessment, Linalool, CAS Registry Number 78-70-6. *Food Chem. Toxicol.* **2015**, *82*, S29–S38, doi:10.1016/j.fct.2015.01.005.
47. Carpentieri, S.; Režek Jambrak, A.; Ferrari, G.; Pataro, G. Pulsed Electric Field-Assisted Extraction of Aroma and Bioactive Compounds From Aromatic Plants and Food By-Products. *Front. Nutr.* **2022**, *8*, doi:10.3389/fnut.2021.792203.
48. Medeiros, C.I.S.; Sousa, M.N.A. de; Filho, G.G.A.; Freitas, F.O.R.; Uchoa, D.P.L.; Nobre, M.S.C.; Bezerra, A.L.D.; Rolim, L. a. D.M.M.; Morais, A.M.B.; Nogueira, T.B.S.S.; et al. Antifungal Activity of Linalool against Fluconazole-Resistant Clinical Strains of Vulvovaginal *Candida Albicans* and Its Predictive Mechanism of Action. *Braz. J. Med. Biol. Res.* **2022**, *55*, e11831, doi:10.1590/1414-431X2022e11831.
49. Rosado-Ramos, R.; Poças, G.M.; Marques, D.; Foito, A.; M. Sevillano, D.; Lopes-da-Silva, M.; Gonçalves, L.G.; Menezes, R.; Ottens, M.; Stewart, D.; et al. Genipin Prevents Alpha-Synuclein Aggregation and Toxicity by Affecting Endocytosis, Metabolism and Lipid Storage. *Nat. Commun.* **2023**, *14*, 1918, doi:10.1038/s41467-023-37561-2.
50. Augustine, E.; Deng, P.; Mou, C.; Okamura, M.; Woolley, B.; Horowitz, M.; Bettinger, C.J. Control Release and Diffusion-Reaction Kinetics of Genipin-Eluting Fibers Using an in Vitro Aneurysm Flow Model. *ACS Biomater. Sci. Eng.* **2021**, *7*, 5144–5153, doi:10.1021/acsbiomaterials.1c00773.
51. Zhao, X.; Song, K.; Wang, S.; Zu, Y.; Li, N.; Yu, X. Micronization of the Pharmaceutically Active Agent Genipin by an Antisolvent Precipitation Process. *Chem. Eng. Technol.* **2013**, *36*, 33–42, doi:10.1002/ceat.201200036.
52. Kalam, M.A.; Alshamsan, A.; Alkholief, M.; Alsarra, I.A.; Ali, R.; Haq, N.; Anwer, M.K.; Shakeel, F. Solubility Measurement and Various Solubility Parameters of Glipizide in Different Neat Solvents. *ACS Omega* **2020**, *5*, 1708–1716, doi:10.1021/acsomega.9b04004.
53. Sun, K.-X.; Chen, Y.-Y.; Li, Z.; Zheng, S.-J.; Wan, W.-J.; Ji, Y.; Hu, K. Genipin Relieves Diabetic Retinopathy by Down-Regulation of Advanced Glycation End Products via the Mitochondrial Metabolism Related Signaling Pathway. *World J. Diabetes* **2023**, *14*, 1349–1368, doi:10.4239/wjd.v14.i9.1349.
54. Ji, Y.-J.; Kim, S.; Kim, J.-J.; Jang, G.Y.; Moon, M.; Kim, H.D. Crude Saponin from Platycodon Grandiflorum Attenuates Aβ-Induced Neurotoxicity via Antioxidant, Anti-Inflammatory and Anti-Apoptotic Signaling Pathways. *Antioxidants* **2021**, *10*, 1968, doi:10.3390/antiox10121968.
55. Jiang, L.; Niu, H.; Chen, Y.; Li, X.; Zhao, Y.; Zhang, C.; Li, M. Quality Control of Platycodon Grandiflorum (Jacq.) A. DC. Based on Value Chains and Food Chain Analysis. *Sci. Rep.* **2023**, *13*, 14048, doi:10.1038/s41598-023-41013-8.
56. Fan, C.; Wang, J.-X.; Xiong, Z.-E.; Hu, S.-S.; Zhou, A.-J.; Yuan, D.; Zhang, C.-C.; Zhou, Z.-Y.; Wang, T. Saponins from Panax Japonicus Improve Neuronal Mitochondrial Injury of Aging Rats. *Pharm. Biol.* **2023**, *61*, 1401–1412, doi:10.1080/13880209.2023.2244532.
57. He, S.; Wang, X.; Chen, J.; Li, X.; Gu, W.; Zhang, F.; Cao, G.; Yu, J. Optimization of the Ultrasonic-Assisted Extraction Technology of Steroidal Saponins from Polygonatum Kingianum Collett & Hemsl and Evaluating Its Quality Planted in Different Areas. *Molecules* **2022**, *27*, 1463, doi:10.3390/molecules27051463.
58. Jiang, X.; Jin, S.; Shao, W.; Zhu, L.; Yan, S.; Lu, J. Saponins of Marsdenia Tenacissima Promotes Apoptosis of Hepatocellular Carcinoma Cells through Damaging Mitochondria Then Activating Cytochrome C/Caspase-9/Caspase-3 Pathway. *J. Cancer* **2022**, *13*, 2855, doi:10.7150/jca.72601.

59. Li, L.; Zhang, W.; Desikan Seshadri, V.D.; Cao, G. Synthesis and Characterization of Gold Nanoparticles from Marsdenia Tenacissima and Its Anticancer Activity of Liver Cancer HepG2 Cells. *Artif. Cells Nanomedicine Biotechnol.* **2019**, *47*, 3029–3036, doi:10.1080/21691401.2019.1642902.
60. Ye, B.; Li, J.; Li, Z.; Yang, J.; Niu, T.; Wang, S. Anti-Tumor Activity and Relative Mechanism of Ethanolic Extract of *Marsdenia Tenacissima* (Asclepiadaceae) against Human Hematologic Neoplasm *in Vitro* and *in Vivo*. *J. Ethnopharmacol.* **2014**, *153*, 258–267, doi:10.1016/j.jep.2014.02.035.
61. Wu, A.-G.; Yong, Y.-Y.; He, C.-L.; Li, Y.-P.; Zhou, X.-Y.; Yu, L.; Chen, Q.; Lan, C.; Liu, J.; Yu, C.-L.; et al. Novel 18-Norspirostane Steroidal Saponins: Extending Lifespan and Mitigating Neurodegeneration through Promotion of Mitophagy and Mitochondrial Biogenesis in *Caenorhabditis Elegans*. *Mech. Ageing Dev.* **2024**, *218*, 111901, doi:10.1016/j.mad.2024.111901.
62. Qiu, W.-Q.; Yu, L.; He, C.-L.; Wu, J.-M.; Law, B.Y.-K.; Yu, C.-L.; Qin, D.-L.; Zhou, X.-G.; Wu, A.-G. Two 18-Norspirostane Steroidal Saponins as Novel Mitophagy Enhancers Improve Alzheimer's Disease. *Clin. Transl. Med.* **2023**, *13*, e1390, doi:10.1002/ctm2.1390.
63. Wang, L.-H.; Song, Y.-T.; Chen, Y.; Cheng, Y.-Y. Solubility of Artemisinin in Ethanol + Water from (278.2 to 343.2) K. *J. Chem. Eng. Data* **2007**, *52*, 757–758, doi:10.1021/je0603426.
64. Ni, X.-C.; Wang, H.-F.; Cai, Y.-Y.; Yang, D.; Alolga, R.N.; Liu, B.; Li, J.; Huang, F.-Q. Ginsenoside Rb1 Inhibits Astrocyte Activation and Promotes Transfer of Astrocytic Mitochondria to Neurons against Ischemic Stroke. *Redox Biol.* **2022**, *54*, 102363, doi:10.1016/j.redox.2022.102363.
65. Vo-An, Q.; Nguyen, T.C.; Nguyen, Q.T.; Vu, Q.T.; Truong, C.D.; Nguyen, T.L.; Ly, T.N.L.; Bach, L.G.; Thai, H. Novel Nanoparticle Biomaterial of Alginate/Chitosan Loading Simultaneously Lovastatin and Ginsenoside RB1: Characteristics, Morphology, and Drug Release Study. *Int. J. Polym. Sci.* **2021**, *2021*, 5214510, doi:10.1155/2021/5214510.
66. Vo-An, Q.; Nguyen, T.C.; Nguyen, Q.T.; Vu, Q.T.; Truong, C.D.; Nguyen, T.L.; Ly, T.N.L.; Bach, L.G.; Thai, H. Novel Nanoparticle Biomaterial of Alginate/Chitosan Loading Simultaneously Lovastatin and Ginsenoside RB1: Characteristics, Morphology, and Drug Release Study. *Int. J. Polym. Sci.* **2021**, *2021*, 5214510, doi:10.1155/2021/5214510.
67. Jiang, L.; Yin, X.; Chen, Y.-H.; Chen, Y.; Jiang, W.; Zheng, H.; Huang, F.-Q.; Liu, B.; Zhou, W.; Qi, L.-W.; et al. Proteomic Analysis Reveals Ginsenoside Rb1 Attenuates Myocardial Ischemia/Reperfusion Injury through Inhibiting ROS Production from Mitochondrial Complex I. *Theranostics* **2021**, *11*, 1703–1720, doi:10.7150/thno.43895.
68. Xia, M.-L.; Xie, X.-H.; Ding, J.-H.; Du, R.-H.; Hu, G. Astragaloside IV Inhibits Astrocyte Senescence: Implication in Parkinson's Disease. *J. Neuroinflammation* **2020**, *17*, 105, doi:10.1186/s12974-020-01791-8.
69. Gu, Y.; Wang, G.; Pan, G.; Fawcett, J.P.; A., J.; Sun, J. Transport and Bioavailability Studies of Astragaloside IV, an Active Ingredient in Radix Astragali. *Basic Clin. Pharmacol. Toxicol.* **2004**, *95*, 295–298, doi:10.1111/j.1742-7843.2004.t01-1-pto950508.x.
70. Xu, L.; Wei, K.; Jiang, J.; Zhang, L. Extraction Optimization of Astragaloside IV by Response Surface Methodology and Evaluation of Its Stability during Sterilization and Storage. *Molecules* **2021**, *26*, 2400, doi:10.3390/molecules26082400.

71. Wang, F.; Zhao, Y.; Chen, S.; Chen, L.; Sun, L.; Cao, M.; Li, C.; Zhou, X. Astragaloside IV Alleviates Ammonia-Induced Apoptosis and Oxidative Stress in Bovine Mammary Epithelial Cells. *Int. J. Mol. Sci.* **2019**, *20*, 600, doi:10.3390/ijms20030600.
72. Zhang, J.; Huang, J.; Lan, J.; Li, Q.; Ke, L.; Jiang, Q.; Li, Y.; Zhang, H.; Zhong, H.; Yang, P.; et al. Astragaloside IV Protects against Autoimmune Myasthenia Gravis in Rats via Regulation of Mitophagy and Apoptosis. *Mol. Med. Rep.* **2024**, *30*, doi:10.3892/mmr.2024.13253.
73. Li, L.; Zou, J.; Zhou, M.; Li, H.; Zhou, T.; Liu, X.; Huang, Q.; Yang, S.; Xiang, Q.; Yu, R. Phenylsulfate-Induced Oxidative Stress and Mitochondrial Dysfunction in Podocytes Are Ameliorated by Astragaloside IV Activation of the SIRT1/PGC1α /Nrf1 Signaling Pathway. *Biomed. Pharmacother.* **2024**, *177*, 117008, doi:10.1016/j.biopha.2024.117008.
74. Luo, Z.; Wang, Y.; Xue, M.; Xia, F.; Zhu, L.; Li, Y.; Jia, D.; Chen, S.; Xu, G.; Lei, Y. Astragaloside IV Ameliorates Fat Metabolism in the Liver of Ageing Mice through Targeting Mitochondrial Activity. *J. Cell. Mol. Med.* **2021**, *25*, 8863–8876, doi:10.1111/jcmm.16847.
75. Ben, Y.; Hao, J.; Zhang, Z.; Xiong, Y.; Zhang, C.; Chang, Y.; Yang, F.; Li, H.; Zhang, T.; Wang, X.; et al. Astragaloside IV Inhibits Mitochondrial-Dependent Apoptosis of the Dorsal Root Ganglion in Diabetic Peripheral Neuropathy Rats Through Modulation of the SIRT1/P53 Signaling Pathway. *Diabetes Metab. Syndr. Obes. Targets Ther.* **2021**, *14*, 1647, doi:10.2147/DMSO.S301068.
76. Ikram, M.; Jo, M.H.; Choe, K.; Khan, A.; Ahmad, S.; Saeed, K.; Kim, M.W.; Kim, M.O. Cycloastragenol, a Triterpenoid Saponin, Regulates Oxidative Stress, Neurotrophic Dysfunctions, Neuroinflammation and Apoptotic Cell Death in Neurodegenerative Conditions. *Cells* **2021**, *10*, 2719, doi:10.3390/cells10102719.
77. Tang, L.; Li, X.; Qin, Y.; Geng, X.; Wang, R.; Tan, W.; Mou, S. The Construction of Oligonucleotide-Cycloastragenol and the Renoprotective Effect Study. *Front. Bioeng. Biotechnol.* **2022**, *10*, doi:10.3389/fbioe.2022.1027517.
78. Yuan, F.; Yang, Y.; Liu, L.; Zhou, P.; Zhu, Y.; Chai, Y.; Chen, K.; Tang, W.; Huang, Q.; Zhang, C. Research Progress on the Mechanism of Astragaloside IV in the Treatment of Asthma. *Heliyon* **2023**, *9*, e22149, doi:10.1016/j.heliyon.2023.e22149.
79. Li, M.; Li, S.; Dou, B.; Zou, Y.; Han, H.; Liu, D.; Ke, Z.; Wang, Z. Cycloastragenol Upregulates SIRT1 Expression, Attenuates Apoptosis and Suppresses Neuroinflammation after Brain Ischemia. *Acta Pharmacol. Sin.* **2020**, *41*, 1025–1032, doi:10.1038/s41401-020-0386-6.
80. Leri, M.; Bertolini, A.; Stefani, M.; Bucciantini, M. EVOO Polyphenols Relieve Synergistically Autophagy Dysregulation in a Cellular Model of Alzheimer's Disease. *Int. J. Mol. Sci.* **2021**, *22*, 7225, doi:10.3390/ijms22137225.
81. Papadimitriou, V.; Sotiroudis, T.G.; Xenakis, A. Olive Oil Microemulsions as a Biomimetic Medium for Enzymatic Studies: Oxidation of Oleuropein. *J. Am. Oil Chem. Soc.* **2005**, *82*, 335–340, doi:10.1007/s11746-005-1075-4.
82. Tasioula-margari, M.; Okogeri, O. Isolation and Characterization of Virgin Olive Oil Phenolic Compounds by HPLC/UV and GC-MS. *J. Food Sci.* **2001**, *66*, 530–534, doi:10.1111/j.1365-2621.2001.tb04597.x.

83. Masrijal, C.D.P.; Harmita, H.; Iskandarsyah, I. IMPROVING TRANSDERMAL DRUG DELIVERY SYSTEM FOR MEDROXYPROGESTERONE ACETATE BY OLIVE OIL AND DIMETHYLSULFOXIDE (DMSO) AS PENETRATION ENHANCERS: IN VITRO PENETRATION STUDY. *Int. J. Pharm. Pharm. Sci.* **2020**, 12–15, doi:10.22159/ijpps.2020v12i4.36762.
84. Madiha, S.; Batool, Z.; Tabassum, S.; Liaquat, L.; Sadir, S.; Shahzad, S.; Naqvi, F.; Saleem, S.; Yousuf, S.; Nawaz, A.; et al. Quercetin Exhibits Potent Antioxidant Activity, Restores Motor and Non-Motor Deficits Induced by Rotenone Toxicity. *PLoS ONE* **2021**, *16*, e0258928, doi:10.1371/journal.pone.0258928.
85. Li, X.; Meng, X.; de Leeuw, T.C.; te Poele, E.M.; Pijning, T.; Dijkhuizen, L.; Liu, W. Enzymatic Glucosylation of Polyphenols Using Glucansucrases and Branching Sucrases of Glycoside Hydrolase Family 70. *Crit. Rev. Food Sci. Nutr.* **2023**, *63*, 5247–5267, doi:10.1080/10408398.2021.2016598.
86. Wisudyaningsih, B.; Setyawan, D.; Siswandono Co-Crystallization of Quercetin and Isonicotinamide Using Solvent Evaporation Method. *Trop. J. Pharm. Res.* **2019**, *18*, 697–702, doi:10.4314/tjpr.v18i4.3.
87. Liu, F.; Peng, B.; Li, M.; Ma, J.; Deng, G.; Zhang, S.; Sheu, W.C.; Zou, P.; Wu, H.; Liu, J.; et al. Targeted Disruption of Tumor Vasculature via Polyphenol Nanoparticles to Improve Brain Cancer Treatment. *Cell Rep. Phys. Sci.* **2022**, *3*, 100691, doi:10.1016/j.xcrp.2021.100691.
88. Wang, W.-W.; Han, R.; He, H.-J.; Li, J.; Chen, S.-Y.; Gu, Y.; Xie, C. Administration of Quercetin Improves Mitochondria Quality Control and Protects the Neurons in 6-OHDA-Lesioned Parkinson's Disease Models. *Aging* **2021**, *13*, 11738–11751, doi:10.18632/aging.202868.
89. Zhao, X.; Wang, C.; Dai, S.; Liu, Y.; Zhang, F.; Peng, C.; Li, Y. Quercetin Protects Ethanol-Induced Hepatocyte Pyroptosis via Scavenging Mitochondrial ROS and Promoting PGC-1α-Regulated Mitochondrial Homeostasis in L02 Cells. *Oxid. Med. Cell. Longev.* **2022**, *2022*, 4591134, doi:10.1155/2022/4591134.
90. Kesh, S.; Kannan, R.R.; Balakrishnan, A. Naringenin Alleviates 6-Hydroxydopamine Induced Parkinsonism in SHSY5Y Cells and Zebrafish Model. *Comp. Biochem. Physiol. Part C Toxicol. Pharmacol.* **2021**, *239*, 108893, doi:10.1016/j.cbpc.2020.108893.
91. Sangpheak, W.; Kicuntod, J.; Schuster, R.; Rungrotmongkol, T.; Wolschann, P.; Kungwan, N.; Viernstein, H.; Mueller, M.; Pongsawasdi, P. Physical Properties and Biological Activities of Hesperetin and Naringenin in Complex with Methylated β-Cyclodextrin. *Beilstein J. Org. Chem.* **2015**, *11*, 2763–2773, doi:10.3762/bjoc.11.297.
92. Du, Y.; Ma, J.; Fan, Y.; Wang, X.; Zheng, S.; Feng, J.; Li, J.; Fan, Z.; Li, G.; Ye, Q. Naringenin: A Promising Therapeutic Agent against Organ Fibrosis. *Oxid. Med. Cell. Longev.* **2021**, *2021*, 1210675, doi:10.1155/2021/1210675.
93. Ahmad, M.H.; Fatima, M.; Ali, M.; Rizvi, M.A.; Mondal, A.C. Naringenin Alleviates Paraquat-Induced Dopaminergic Neuronal Loss in SH-SY5Y Cells and a Rat Model of Parkinson's Disease. *Neuropharmacology* **2021**, *201*, 108831, doi:10.1016/j.neuropharm.2021.108831.
94. Lu, W.; Yu, C.R.; Lien, H.; Sheu, G.; Cherng, S. Cytotoxicity of Naringenin Induces Bax-mediated Mitochondrial Apoptosis in Human Lung Adenocarcinoma A549 Cells. *Environ. Toxicol.* **2020**, *35*, 1386–1394, doi:10.1002/tox.23003.
95. Chen, G.; Zeng, L.; Yan, F.; Liu, J.; Qin, M.; Wang, F.; Zhang, X. Long-Term Oral Administration of Naringenin Counteracts Aging-Related Retinal Degeneration via Regulation of Mitochondrial Dynamics and Autophagy. *Front. Pharmacol.* **2022**, *13*, 919905, doi:10.3389/fphar.2022.919905.

96. Cho, I.; Song, H.; Cho, J.H. Flavonoids Mitigate Neurodegeneration in Aged Caenorhabditis Elegans by Mitochondrial Uncoupling. *Food Sci. Nutr.* **2020**, *8*, 6633–6642, doi:10.1002/fsn3.1956.
97. Goujon, M.; Liang, Z.; Soriano-Castell, D.; Currais, A.; Maher, P. The Neuroprotective Flavonoids Sterubin and Fisetin Maintain Mitochondrial Health under Oxytotic/Ferroptotic Stress and Improve Bioenergetic Efficiency in HT22 Neuronal Cells. *Antioxidants* **2024**, *13*, 460, doi:10.3390/antiox13040460.
98. Bécquer-Viart, M.Á.; Armentero-López, A.; Alvarez-Almiñaque, D.; Fernández-Acosta, R.; Matos-Peralta, Y.; D'Vries, R.F.; Marín-Prida, J.; Pardo-Andreu, G.L. Gossypitrin, A Naturally Occurring Flavonoid, Attenuates Iron-Induced Neuronal and Mitochondrial Damage. *Molecules* **2021**, *26*, 3364, doi:10.3390/molecules26113364.
99. González, J.; Cuéllar, A.; Nossin, E.; Monan, M. Iron Chelating Activity of Gossypitrin Isolated from the Petals of Talipariti Elatum Sw. (Fryxell) Malvaceae. *J. Agric. Stud.* **2017**, *5*, 1–12, doi:10.5296/jas.v5i2.11174.
100. Huang, M.; Singh, N.; Kainth, R.; Khalid, M.; Kushwah, A.S.; Kumar, M. Mechanistic Insight into Diosmin-Induced Neuroprotection and Memory Improvement in Intracerebroventricular-Quinolinic Acid Rat Model: Resurrection of Mitochondrial Functions and Antioxidants. *Evid.-Based Complement. Altern. Med. ECAM* **2022**, *2022*, doi:10.1155/2022/8584558.
101. Zingale, E.; Rizzo, S.; Bonaccorso, A.; Consoli, V.; Vanella, L.; Musumeci, T.; Spadaro, A.; Pignatello, R. Optimization of Lipid Nanoparticles by Response Surface Methodology to Improve the Ocular Delivery of Diosmin: Characterization and In-Vitro Anti-Inflammatory Assessment. *Pharmaceutics* **2022**, *14*, 1961, doi:10.3390/pharmaceutics14091961.
102. Pielorz, S.; Węglińska, M.; Mazurek, S.; Szostak, R. Quantitative Determination of Diosmin in Tablets by Infrared and Raman Spectroscopy. *Molecules* **2022**, *27*, 8276, doi:10.3390/molecules27238276.
103. Liu, X.; Liu, W.; Wang, C.; Chen, Y.; Liu, P.; Hayashi, T.; Mizuno, K.; Hattori, S.; Fujisaki, H.; Ikejima, T. Silibinin Attenuates Motor Dysfunction in a Mouse Model of Parkinson's Disease by Suppression of Oxidative Stress and Neuroinflammation along with Promotion of Mitophagy. *Physiol. Behav.* **2021**, *239*, 113510, doi:10.1016/j.physbeh.2021.113510.
104. Saller, R.; Melzer, J.; Reichling, J.; Brignoli, R.; Meier, R. An Updated Systematic Review of the Pharmacology of Silymarin. *Forsch. Komplementärmedizin Res. Complement. Med.* **2007**, *14*, 70–80, doi:10.1159/000100581.
105. Kadoglou, N.P.E.; Panayiotou, C.; Vardas, M.; Balaskas, N.; Kostomitsopoulos, N.G.; Tsaroucha, A.K.; Valsami, G. A Comprehensive Review of the Cardiovascular Protective Properties of Silibinin/Silymarin: A New Kid on the Block. *Pharmaceuticals* **2022**, *15*, 538, doi:10.3390/ph15050538.
106. Liu, X.; Wang, C.; Liu, W.; Song, S.; Fu, J.; Hayashi, T.; Mizuno, K.; Hattori, S.; Fujisaki, H.; Ikejima, T. Oral Administration of Silibinin Ameliorates Cognitive Deficits of Parkinson's Disease Mouse Model by Restoring Mitochondrial Disorders in Hippocampus. *Neurochem. Res.* **2021**, *46*, 2317–2332, doi:10.1007/s11064-021-03363-5.

107. Tie, F.; Fu, Y.; Hu, N.; Wang, H. Silibinin Protects against H2O2-Induced Oxidative Damage in SH-SY5Y Cells by Improving Mitochondrial Function. *Antioxidants* **2022**, *11*, 1101, doi:10.3390/antiox11061101.
108. Esselun, C.; Bruns, B.; Hagl, S.; Grewal, R.; Eckert, G.P. Impact of Silibinin A on Bioenergetics in PC12APPsw Cells and Mitochondrial Membrane Properties in Murine Brain Mitochondria. *Antioxidants* **2021**, *10*, 1520, doi:10.3390/antiox10101520.
109. Iyengar, R.M.; Devaraj, E. Silibinin Triggers the Mitochondrial Pathway of Apoptosis in Human Oral Squamous Carcinoma Cells. *Asian Pac. J. Cancer Prev. APJCP* **2020**, *21*, 1877–1882, doi:10.31557/APJCP.2020.21.7.1877.
110. Naia, L.; Pinho, C.M.; Dentoni, G.; Liu, J.; Leal, N.S.; Ferreira, D.M.S.; Schreiner, B.; Filadi, R.; Fão, L.; Connolly, N.M.C.; et al. Neuronal Cell-Based High-Throughput Screen for Enhancers of Mitochondrial Function Reveals Luteolin as a Modulator of Mitochondria-Endoplasmic Reticulum Coupling. *BMC Biol.* **2021**, *19*, 57, doi:10.1186/s12915-021-00979-5.
111. Sato, A.; Shinozaki, N.; Tamura, H. Secoiridoid Type of Antiallergic Substances in Olive Waste Materials of Three Japanese Varieties of Olea Europaea. *J. Agric. Food Chem.* **2014**, *62*, 7787–7795, doi:10.1021/jf502151b.
112. Xu, H.; Yu, W.; Sun, S.; Li, C.; Zhang, Y.; Ren, J. Luteolin Attenuates Doxorubicin-Induced Cardiotoxicity Through Promoting Mitochondrial Autophagy. *Front. Physiol.* **2020**, *11*, 113, doi:10.3389/fphys.2020.00113.
113. Ma, J.; Pan, Z.; Du, H.; Chen, X.; Zhu, X.; Hao, W.; Zheng, Q.; Tang, X. Luteolin Induces Apoptosis by Impairing Mitochondrial Function and Targeting the Intrinsic Apoptosis Pathway in Gastric Cancer Cells. *Oncol. Lett.* **2023**, *26*, 327, doi:10.3892/ol.2023.13913.
114. Chen, D.; Shen, F.; Liu, J.; Tang, H.; Zhang, K.; Teng, X.; Yang, F. The Protective Effect of Luteolin on Chicken Spleen Lymphocytes from Ammonia Poisoning through Mitochondria and Balancing Energy Metabolism Disorders. *Poult. Sci.* **2023**, *102*, 103093, doi:10.1016/j.psj.2023.103093.
115. Cirmi, S.; Maugeri, A.; Lombardo, G.E.; Russo, C.; Musumeci, L.; Gangemi, S.; Calapai, G.; Barreca, D.; Navarra, M. A Flavonoid-Rich Extract of Mandarin Juice Counteracts 6-OHDA-Induced Oxidative Stress in SH-SY5Y Cells and Modulates Parkinson-Related Genes. *Antioxidants* **2021**, *10*, 539, doi:10.3390/antiox10040539.
116. Safdar, M.N.; Kausar, T.; Jabbar, S.; Mumtaz, A.; Ahad, K.; Saddozai, A.A. Extraction and Quantification of Polyphenols from Kinnow (*Citrus Reticulate* L.) Peel Using Ultrasound and Maceration Techniques. *J. Food Drug Anal.* **2017**, *25*, 488–500, doi:10.1016/j.jfda.2016.07.010.
117. Antunes, M.S.; Ladd, F.V.L.; Ladd, A.A.B.L.; Moreira, A.L.; Boeira, S.P.; Cattelan Souza, L. Hesperidin Protects against Behavioral Alterations and Loss of Dopaminergic Neurons in 6-OHDA-Lesioned Mice: The Role of Mitochondrial Dysfunction and Apoptosis. *Metab. Brain Dis.* **2021**, *36*, 153–167, doi:10.1007/s11011-020-00618-y.
118. Majumdar, S.; Srirangam, R. Solubility, Stability, Physicochemical Characteristics and In Vitro Ocular Tissue Permeability of Hesperidin: A Natural Bioflavonoid. *Pharm. Res.* **2009**, *26*, 1217–1225, doi:10.1007/s11095-008-9729-6.

119. Bisen, A.C.; Rawat, P.; Sharma, G.; Sanap, S.N.; Agrawal, S.; Kumar, S.; Kumar, A.; Choudhury, A.D.; Kamboj, S.; Narender, T.; et al. Hesperidin: Enrichment, Forced Degradation, and Structural Elucidation of Potential Degradation Products Using Spectral Techniques. *Rapid Commun. Mass Spectrom.* **2023**, *37*, e9615, doi:10.1002/rcm.9615.
120. Lv, Y.; Liu, Z.; Deng, L.; Xia, S.; Mu, Q.; Xiao, B.; Xiu, Y.; Liu, Z. Hesperetin Promotes Bladder Cancer Cells Death via the PI3K/AKT Pathway by Network Pharmacology and Molecular Docking. *Sci. Rep.* **2024**, *14*, 1009, doi:10.1038/s41598-023-50476-8.
121. Liu, L.; Chen, J. Solubility of Hesperetin in Various Solvents from (288.2 to 323.2) K. *J. Chem. Eng. Data* **2008**, *53*, 1649–1650, doi:10.1021/je800078j.
122. Bisen, A.C.; Rawat, P.; Sharma, G.; Sanap, S.N.; Agrawal, S.; Kumar, S.; Kumar, A.; Choudhury, A.D.; Kamboj, S.; Narender, T.; et al. Hesperidin: Enrichment, Forced Degradation, and Structural Elucidation of Potential Degradation Products Using Spectral Techniques. *Rapid Commun. Mass Spectrom.* **2023**, *37*, e9615, doi:10.1002/rcm.9615.
123. Li, W.; Cai, Z.; Schindler, F.; Afjehi-Sadat, L.; Montsch, B.; Heffeter, P.; Heiss, E.H.; Weckwerth, W. Elevated PINK1/Parkin-Dependent Mitophagy and Boosted Mitochondrial Function Mediate Protection of HepG2 Cells from Excess Palmitic Acid by Hesperetin. *J. Agric. Food Chem.* **2024**, *72*, 13039–13053, doi:10.1021/acs.jafc.3c09132.
124. Park, D.H.; Park, J.Y.; Kang, K.S.; Hwang, G.S. Neuroprotective Effect of Gallocatechin Gallate on Glutamate-Induced Oxidative Stress in Hippocampal HT22 Cells. *Molecules* **2021**, *26*, 1387, doi:10.3390/molecules26051387.
125. Sharma, S.K.; Bhatt, P. Controlled Release of Bi-Layered EGCG Tablets Using 3D Printing Techniques. *J. Pharm. Res. Int.* **2020**, 5–13, doi:10.9734/jpri/2020/v32i3931019.
126. Hu, S.; Wang, T.; Ni, L.; Hu, F.; Yue, B.; Zheng, Y.; Wang, T.; Kumar, A.; Wang, Y.; Wang, J.; et al. Icariin Ameliorates D-Galactose-Induced Cell Injury in Neuron-like PC12 Cells by Inhibiting MPTP Opening. *Curr. Med. Sci.* **2024**, *44*, 748–758, doi:10.1007/s11596-024-2892-0.
127. Li, Y.; Sun, S.; Chang, Q.; Zhang, L.; Wang, G.; Chen, W.; Miao, X.; Zheng, Y. A Strategy for the Improvement of the Bioavailability and Antiosteoporosis Activity of BCS IV Flavonoid Glycosides through the Formulation of Their Lipophilic Aglycone into Nanocrystals. *Mol. Pharm.* **2013**, *10*, 2534–2542, doi:10.1021/mp300688t.
128. Wu, Y.-T.; Lin, C.-W.; Lin, L.-C.; Chiu, A.W.; Chen, K.-K.; Tsai, T.-H. Analysis of Biliary Excretion of Icariin in Rats. *J. Agric. Food Chem.* **2010**, *58*, 9905–9911, doi:10.1021/jf101987j.
129. Song, L.; Chen, X.; Mi, L.; Liu, C.; Zhu, S.; Yang, T.; Luo, X.; Zhang, Q.; Lu, H.; Liang, X. Icariin-induced Inhibition of SIRT6/NF-κB Triggers Redox Mediated Apoptosis and Enhances Anti-tumor Immunity in Triple-negative Breast Cancer. *Cancer Sci.* **2020**, *111*, 4242–4256, doi:10.1111/cas.14648.
130. Hua, W.; Li, S.; Luo, R.; Wu, X.; Zhang, Y.; Liao, Z.; Song, Y.; Wang, K.; Zhao, K.; Yang, S.; et al. Icariin Protects Human Nucleus Pulposus Cells from Hydrogen Peroxide-Induced Mitochondria-Mediated Apoptosis by Activating Nuclear Factor Erythroid 2-Related Factor 2. *Biochim. Biophys. Acta BBA - Mol. Basis Dis.* **2020**, *1866*, 165575, doi:10.1016/j.bbadis.2019.165575.

131. Wang, N.; Wang, H.; Pan, Q.; Kang, J.; Liang, Z.; Zhang, R. The Combination of β-Asarone and Icariin Inhibits Amyloid-β and Reverses Cognitive Deficits by Promoting Mitophagy in Models of Alzheimer's Disease. *Oxid. Med. Cell. Longev.* **2021**, *2021*, 7158444, doi:10.1155/2021/7158444.
132. Wang, N.; Wang, H.; Li, L.; Li, Y.; Zhang, R. β-Asarone Inhibits Amyloid-β by Promoting Autophagy in a Cell Model of Alzheimer's Disease. *Front. Pharmacol.* **2020**, *10*, doi:10.3389/fphar.2019.01529.
133. Ramalingam, P.; Ganesan, P.; Prabakaran, D.S.; Gupta, P.K.; Jonnalagadda, S.; Govindarajan, K.; Vishnu, R.; Sivalingam, K.; Sodha, S.; Choi, D.-K.; et al. Lipid Nanoparticles Improve the Uptake of α-Asarone Into the Brain Parenchyma: Formulation, Characterization, In Vivo Pharmacokinetics, and Brain Delivery. *AAPS PharmSciTech* **2020**, *21*, 299, doi:10.1208/s12249-020-01832-8.
134. Unger, P.; Melzig, M.F. Comparative Study of the Cytotoxicity and Genotoxicity of Alpha- and Beta-Asarone. *Sci. Pharm.* **2012**, *80*, 663–668, doi:10.3797/scipharm.1204-21.
135. Yu, J.; Gao, X.; Zhang, L.; Shi, H.; Yan, Y.; Han, Y.; Wu, C.; Liu, Y.; Fang, M.; Huang, C.; et al. Magnolol Extends Lifespan and Improves Age-Related Neurodegeneration in Caenorhabditis Elegans via Increase of Stress Resistance. *Sci. Rep.* **2024**, *14*, 3158, doi:10.1038/s41598-024-53374-9.
136. Usach, I.; Alaimo, A.; Fernández, J.; Ambrosini, A.; Mocini, S.; Ochiuz, L.; Peris, J.-E. Magnolol and Honokiol: Two Natural Compounds with Similar Chemical Structure but Different Physicochemical and Stability Properties. *Pharmaceutics* **2021**, *13*, 224, doi:10.3390/pharmaceutics13020224.
137. Li, Y.-F.; Zhu, B.-W.; Chen, T.; Chen, L.-H.; Wu, D.; Hu, J.-N. Construction of Magnolol Nanoparticles for Alleviation of Ethanol-Induced Acute Gastric Injury. *J. Agric. Food Chem.* **2024**, *72*, 7933–7942, doi:10.1021/acs.jafc.3c09902.
138. Liu, Z.; Zhang, H.; Wang, H.; Wei, L.; Niu, L. Magnolol Alleviates IL-1β-Induced Dysfunction of Chondrocytes Through Repression of SIRT1/AMPK/PGC-1α Signaling Pathway. *J. Interferon Cytokine Res.* **2020**, *40*, 145–151, doi:10.1089/jir.2019.0139.
139. Cheng, G.; Hardy, M.; Zielonka, J.; Weh, K.; Zielonka, M.; Boyle, K.A.; Abu Eid, M.; McAllister, D.; Bennett, B.; Kresty, L.A.; et al. Mitochondria-Targeted Magnolol Inhibits OXPHOS, Proliferation, and Tumor Growth via Modulation of Energetics and Autophagy in Melanoma Cells. *Cancer Treat. Res. Commun.* **2020**, *25*, 100210, doi:10.1016/j.ctarc.2020.100210.
140. Jin, Y.C.; Kim, K.J.; Kim, Y.M.; Ha, Y.M.; Kim, H.J.; Yun, U.J.; Bae, K.H.; Kim, Y.S.; Kang, S.S.; Seo, H.G.; et al. Anti-Apoptotic Effect of Magnolol in Myocardial Ischemia and Reperfusion Injury Requires Extracellular Signal-Regulated Kinase1/2 Pathways in Rat In Vivo. *Exp. Biol. Med.* **2008**, *233*, 1280–1288, doi:10.3181/0803-RM-79.
141. Tao, C.; Chen, J.; Huang, X.; Chen, Z.; Li, X.; Li, Y.; Xu, Y.; Ma, M.; Wu, Z. CT1-3, a Novel Magnolol-Sulforaphane Hybrid Suppresses Tumorigenesis through Inducing Mitochondria-Mediated Apoptosis and Inhibiting Epithelial Mesenchymal Transition. *Eur. J. Med. Chem.* **2020**, *199*, 112441, doi:10.1016/j.ejmech.2020.112441.
142. Santonocito, D.; Vivero-Lopez, M.; Lauro, M.R.; Torrisi, C.; Castelli, F.; Sarpietro, M.G.; Puglia, C. Design of Nanotechnological Carriers for Ocular Delivery of Mangiferin: Preformulation Study. *Molecules* **2022**, *27*, 1328, doi:10.3390/molecules27041328.

143. Azimullah, S.; Meeran, M.F.N.; Ayoob, K.; Arunachalam, S.; Ojha, S.; Beiram, R. Tannic Acid Mitigates Rotenone-Induced Dopaminergic Neurodegeneration by Inhibiting Inflammation, Oxidative Stress, Apoptosis, and Glutamate Toxicity in Rats. *Int. J. Mol. Sci.* **2023**, *24*, 9876, doi:10.3390/ijms24129876.
144. Kruthika, N.L.; Raju, G.B.; Prabhakar, S. Degradation of Tannic Acid Powered by TiO2 Nanoparticles. *Mater. Sci. Forum* **2013**, *734*, 117–126, doi:10.4028/www.scientific.net/MSF.734.117.
145. Salman, Mohd.; Tabassum, H.; Parvez, S. Tannic Acid Provides Neuroprotective Effects Against Traumatic Brain Injury Through the PGC-1α/Nrf2/HO-1 Pathway. *Mol. Neurobiol.* **2020**, *57*, 2870–2885, doi:10.1007/s12035-020-01924-3.
146. Li, C.-C.; Tsai, B.C.-K.; Annseles Rajula, S.; Hsu, C.-H.; Chen, M.-C.; Kuo, C.-H.; Yeh, C.-M.; Hsieh, D.J.-Y.; Kuo, W.-W.; Huang, C.-Y. Tannic Acid Impedes the Proliferation of Bladder Cancer Cells by Elevating Mitochondrial Pathways of Apoptosis. *Cell Biochem. Biophys.* **2024**, *82*, 1325–1333, doi:10.1007/s12013-024-01286-w.
147. Zeng, Y.; Cao, G.; Lin, L.; Zhang, Y.; Luo, X.; Ma, X.; Aiyisake, A.; Cheng, Q. Resveratrol Attenuates Sepsis-Induced Cardiomyopathy in Rats through Anti-Ferroptosis via the Sirt1/Nrf2 Pathway. *J. Investig. Surg. Off. J. Acad. Surg. Res.* **2023**, *36*, doi:10.1080/08941939.2022.2157521.
148. Delmas, D.; Aires, V.; Limagne, E.; Dutartre, P.; Mazué, F.; Ghiringhelli, F.; Latruffe, N. Transport, Stability, and Biological Activity of Resveratrol. *Ann. N. Y. Acad. Sci.* **2011**, *1215*, 48–59, doi:10.1111/j.1749-6632.2010.05871.x.
149. Zheng, M.; Bai, Y.; Sun, X.; Fu, R.; Liu, L.; Liu, M.; Li, Z.; Huang, X. Resveratrol Reestablishes Mitochondrial Quality Control in Myocardial Ischemia/Reperfusion Injury through Sirt1/Sirt3-Mfn2-Parkin-PGC-1α Pathway. *Molecules* **2022**, *27*, 5545, doi:10.3390/molecules27175545.
150. Wu, L.; Chen, Q.; Dong, B.; Geng, H.; Wang, Y.; Han, D.; Zhu, X.; Liu, H.; Zhang, Z.; Yang, Y.; et al. Resveratrol Alleviates Lipopolysaccharide-Induced Liver Injury by Inducing SIRT1/P62-Mediated Mitophagy in Gibel Carp (Carassius Gibelio). *Front. Immunol.* **2023**, *14*, 1177140, doi:10.3389/fimmu.2023.1177140.
151. Zhao, Q.; Tian, Z.; Zhou, G.; Niu, Q.; Chen, J.; Li, P.; Dong, L.; Xia, T.; Zhang, S.; Wang, A. SIRT1-Dependent Mitochondrial Biogenesis Supports Therapeutic Effects of Resveratrol against Neurodevelopment Damage by Fluoride. *Theranostics* **2020**, *10*, 4822–4838, doi:10.7150/thno.42387.
152. Adedara, A.O.; Babalola, A.D.; Stephano, F.; Awogbindin, I.O.; Olopade, J.O.; Rocha, J.B.T.; Whitworth, A.J.; Abolaji, A.O. An Assessment of the Rescue Action of Resveratrol in Parkin Loss of Function-Induced Oxidative Stress in Drosophila Melanogaster. *Sci. Rep.* **2022**, *12*, 3922, doi:10.1038/s41598-022-07909-7.
153. Fu, Y.; Ye, Y.; Zhu, G.; Xu, Y.; Sun, J.; Wu, H.; Feng, F.; Wen, Z.; Jiang, S.; Li, Y.; et al. Resveratrol Induces Human Colorectal Cancer Cell Apoptosis by Activating the Mitochondrial Pathway via Increasing Reactive Oxygen Species. *Mol. Med. Rep.* **2021**, *23*, 1–1, doi:10.3892/mmr.2020.11809.
154. Yan, W.; Ren, D.; Feng, X.; Huang, J.; Wang, D.; Li, T.; Zhang, D. Neuroprotective and Anti-Inflammatory Effect of Pterostilbene Against Cerebral Ischemia/Reperfusion Injury via Suppression of COX-2. *Front. Pharmacol.* **2021**, *12*, 770329, doi:10.3389/fphar.2021.770329.
155. Tran, T.M.; Atanasova, V.; Tardif, C.; Richard-Forget, F. Stilbenoids as Promising Natural Product-Based Solutions in a Race against Mycotoxigenic Fungi: A Comprehensive Review. *J. Agric. Food Chem.* **2023**, *71*, 5075–5092, doi:10.1021/acs.jafc.3c00407.

156. Wu, Y.; Hu, Q.; Wang, X.; Cheng, H.; Yu, J.; Li, Y.; Luo, J.; Zhang, Q.; Wu, J.; Zhang, G. Pterostilbene Attenuates Microglial Inflammation and Brain Injury after Intracerebral Hemorrhage in an OPA1-Dependent Manner. *Front. Immunol.* **2023**, *14*, 1172334, doi:10.3389/fimmu.2023.1172334.
157. Gao, H.; Liu, Z.; Xu, W.; Wang, Q.; Zhang, C.; Ding, Y.; Nie, W.; Lai, J.; Chen, Y.; Huang, H. Pterostilbene Promotes Mitochondrial Apoptosis and Inhibits Proliferation in Glioma Cells. *Sci. Rep.* **2021**, *11*, 6381, doi:10.1038/s41598-021-85908-w.
158. Tang, Z.; Lai, C.; Luo, J.; Ding, Y.; Chen, Q.; Guan, Z. Mangiferin Prevents the Impairment of Mitochondrial Dynamics and an Increase in Oxidative Stress Caused by Excessive Fluoride in SH-SY5Y Cells. *J. Biochem. Mol. Toxicol.* **2021**, *35*, e22705, doi:10.1002/jbt.22705.
159. Gerber, G.; Fox, L.; Gerber, M.; Preez, J.; van Zyl, S.; Boneschans, B.; Plessis, J. Stability, Clinical Efficacy, and Antioxidant Properties of Honeybush Extracts in Semi-Solid Formulations. *Pharmacogn. Mag.* **2015**, *11*, s337–s351, doi:10.4103/0973-1296.166063.
160. Purnomo, Y.; Soeatmadji, D.W.; Sumitro, S.B.; Widodo, M.A. Anti-Diabetic Potential of *Urena Lobata* Leaf Extract through Inhibition of Dipeptidyl Peptidase IV Activity. *Asian Pac. J. Trop. Biomed.* **2015**, *5*, 645–649, doi:10.1016/j.apjtb.2015.05.014.
161. Wang, Z.; Wang, R.; Tian, J.; Zhao, B.; Wei, X.-F.; Su, Y.-L.; Li, C.-Y.; Cao, S.-G.; Ji, T.-F.; Wang, L. The Effect of Ultrasound on Lipase-Catalyzed Regioselective Acylation of Mangiferin in Non-Aqueous Solvents: Original Article. *J. Asian Nat. Prod. Res.* **2010**, *12*, 56–63, doi:10.1080/10286020903431080.
162. Park, C.; Cha, H.-J.; Hwangbo, H.; Bang, E.; Kim, H.-S.; Yun, S.J.; Moon, S.-K.; Kim, W.-J.; Kim, G.-Y.; Lee, S.-O.; et al. Activation of Heme Oxygenase-1 by Mangiferin in Human Retinal Pigment Epithelial Cells Contributes to Blocking Oxidative Damage. *Biomol. Ther.* **2024**, *32*, 329–340, doi:10.4062/biomolther.2023.175.
163. Wang, X.-L.; Feng, S.-T.; Wang, Y.-T.; Zhang, N.-N.; Guo, Z.-Y.; Yan, X.; Yuan, Y.-H.; Wang, Z.-Z.; Chen, N.-H.; Zhang, Y. Mangiferin, a Natural Glucoxilxanthone, Inhibits Mitochondrial Dynamin-Related Protein 1 and Relieves Aberrant Mitophagic Proteins in Mice Model of Parkinson's Disease. *Phytomedicine* **2022**, *104*, 154281, doi:10.1016/j.phymed.2022.154281.
164. Yu, H.; Hou, G.; Cao, J.; Yin, Y.; Zhao, Y.; Cheng, L. Mangiferin Alleviates Mitochondrial ROS in Nucleus Pulposus Cells and Protects against Intervertebral Disc Degeneration via Suppression of NF-κB Signaling Pathway. *Oxid. Med. Cell. Longev.* **2021**, *2021*, 6632786, doi:10.1155/2021/6632786.
165. Esselun, C.; Theyssen, E.; Eckert, G.P. Effects of Urolithin A on Mitochondrial Parameters in a Cellular Model of Early Alzheimer Disease. *Int. J. Mol. Sci.* **2021**, *22*, 8333, doi:10.3390/ijms22158333.
166. Lessard-Lord, J.; Plante, P.-L.; Desjardins, Y. Purified Recombinant Enzymes Efficiently Hydrolyze Conjugated Urinary (Poly)Phenol Metabolites. *Food Funct.* **2022**, *13*, 10895–10911, doi:10.1039/D2FO02229J.
167. Cho, S.I.; Jo, E.-R.; Song, H. Urolithin A Attenuates Auditory Cell Senescence by Activating Mitophagy. *Sci. Rep.* **2022**, *12*, 7704, doi:10.1038/s41598-022-11894-2.
168. D'Amico, D.; Olmer, M.; Fouassier, A.M.; Valdés, P.; Andreux, P.A.; Rinsch, C.; Lotz, M. Urolithin A Improves Mitochondrial Health, Reduces Cartilage Degeneration, and Alleviates Pain in Osteoarthritis. *Aging Cell* **2022**, *21*, e13662, doi:10.1111/acel.13662.

169. Singh, A.; D'Amico, D.; Andreux, P.A.; Fouassier, A.M.; Blanco-Bose, W.; Evans, M.; Aebischer, P.; Auwerx, J.; Rinsch, C. Urolithin A Improves Muscle Strength, Exercise Performance, and Biomarkers of Mitochondrial Health in a Randomized Trial in Middle-Aged Adults. *Cell Rep. Med.* **2022**, *3*, 100633, doi:10.1016/j.xcrm.2022.100633.
170. Kshirsagar, S.; Alvir, R.V.; Pradeepkiran, J.A.; Hindle, A.; Vijayan, M.; Ramasubramaniam, B.; Kumar, S.; Reddy, A.P.; Reddy, P.H. A Combination Therapy of Urolithin A+EGCG Has Stronger Protective Effects than Single Drug Urolithin A in a Humanized Amyloid Beta Knockin Mice for Late-Onset Alzheimer's Disease. *Cells* **2022**, *11*, 2660, doi:10.3390/cells11172660.
171. Brasil, F.B.; de Almeida, F.J.S.; Luckachaki, M.D.; Dall'Oglio, E.L.; de Oliveira, M.R. The Isothiocyanate Sulforaphane Prevents Mitochondrial Impairment and Neuroinflammation in the Human Dopaminergic SH-SY5Y and in the Mouse Microglial BV2 Cells: Role for Heme Oxygenase-1. *Metab. Brain Dis.* **2023**, *38*, 419–435, doi:10.1007/s11011-022-00990-x.
172. Sim, H.W.; Lee, W.-Y.; Lee, R.; Yang, S.Y.; Ham, Y.-K.; Lim, S.D.; Park, H.-J. The Anti-Inflammatory Effects of Broccoli (Brassica Oleracea L. Var. Italica) Sprout Extract in RAW 264.7 Macrophages and a Lipopolysaccharide-Induced Liver Injury Model. *Curr. Issues Mol. Biol.* **2023**, *45*, 9117–9131, doi:10.3390/cimb45110572.
173. Folbergrová, J.; Ješina, P.; Otáhal, J. Protective Effect of Sulforaphane on Oxidative Stress and Mitochondrial Dysfunction Associated with Status Epilepticus in Immature Rats. *Mol. Neurobiol.* **2023**, *60*, 2024–2035, doi:10.1007/s12035-022-03201-x.
174. Napoli, E.; Flores, A.; Mansuri, Y.; Hagerman, R.J.; Giulivi, C. Sulforaphane Improves Mitochondrial Metabolism in Fibroblasts from Patients with Fragile X-Associated Tremor and Ataxia Syndrome. *Neurobiol. Dis.* **2021**, *157*, 105427, doi:10.1016/j.nbd.2021.105427.
175. Anjum, R.; Raza, C.; Faheem, M.; Ullah, A.; Chaudhry, M. Neuroprotective Potential of Mentha Piperita Extract Prevents Motor Dysfunctions in Mouse Model of Parkinson's Disease through Anti-Oxidant Capacities. *PLOS ONE* **2024**, *19*, e0302102, doi:10.1371/journal.pone.0302102.
176. Singh, M.; Jha, A.; Kumar, A.; Hettiarachchy, N.; Rai, A.K.; Sharma, D. Influence of the Solvents on the Extraction of Major Phenolic Compounds (Punicalagin, Ellagic Acid and Gallic Acid) and Their Antioxidant Activities in Pomegranate Aril. *J. Food Sci. Technol.* **2014**, *51*, 2070–2077, doi:10.1007/s13197-014-1267-0.
177. Liang, Z.; Soriano-Castell, D.; Kepchia, D.; Duggan, B.M.; Currais, A.; Schubert, D.; Maher, P. Cannabinol Inhibits Oxytosis/Ferroptosis by Directly Targeting Mitochondria Independently of Cannabinoid Receptors. *Free Radic. Biol. Med.* **2022**, *180*, 33–51, doi:10.1016/j.freeradbiomed.2022.01.001.
178. Tavčar, E.; Vidak, M. Experimental Investigation and Thermodynamic Modelling of Cannabidiol and Curcumin in Different Solvents. *J. Mol. Liq.* **2024**, *410*, 125511, doi:10.1016/j.molliq.2024.125511.
